# Animal-free matrix alternative for three-dimensional *in vitro* angiogenesis models

**DOI:** 10.3389/ftox.2026.1768268

**Published:** 2026-06-18

**Authors:** Elle Koivunotko, Chris S. Pridgeon, Lauri Paasonen, Riina Harjumäki

**Affiliations:** 1 Division of Pharmaceutical Biosciences, Drug Research Program, Faculty of Pharmacy, University of Helsinki, Helsinki, Finland; 2 UPM Biomedicals, UPM-Kymmene Corporation, Helsinki, Finland

**Keywords:** angiogenesis, animal-free, capillary-like formation, coculture, *in vitro* 3D cell model, nanofibrillated cellulose hydrogel

## Abstract

All metabolically active tissues have a dense vascular network to supply oxygen and nutrients. Angiogenesis, the formation of new blood vessels from existing ones, can be activated by the lack of oxygen in cells, disease, trauma, or tumor growth. Due to its essential role in cell survival, angiogenesis has been extensively studied, and therapeutic control of this process is of growing interest. Simultaneously, research aims to find and standardize non-animal testing methods for future use. However, the establishment of reproducible and physiologically relevant vascular networks *in vitro* is challenging, particularly due to the widespread use of heterogeneous animal-derived matrices which are not in keeping with the principles of the 3Rs (Replacement, Reduction, Refinement) in science and research. To address these limitations and support the development of ethical and sustainable *in vitro* methods, we present a novel animal-free, three-dimensional angiogenesis model based on medical grade plant-derived nanofibrillated cellulose hydrogel (NFCh). The model uses human umbilical vein endothelial cells (HUVECs) and human adipose-derived stromal cells (hASCs) cultured in medium supplemented with human serum and NFCh matrix. First, HUVECs were stimulated with stem cell-conditioned medium and cultured in varying NFCh concentrations (0.125%–2.4%) tuned to identify the optimal matrix stiffness for capillary-like structure formation. HUVECs were then cocultured with hASCs in the best-performing hydrogel concentration, followed by imaging and proteomics analysis. We observed formation of capillary-like structures, comparable to the most commonly used animal-derived extracellular matrix (ECM) control. Interestingly cells in lower NFCh concentration (0.125%) had higher expression of wound healing and angiogenesis-related proteins while higher stiffnesses (1.5%) had higher ECM-related protein expression. Importantly, vascular network morphology and organization could be adjusted by modifying only NFCh concentration. These findings demonstrate the potential of NFCh as a reproducible, animal-free alternative to conventional angiogenesis matrices with relevance to regenerative therapies, toxicological testing, drug screening, and the development of a 3Rs-compliant *in vitro* system.

## Graphical Abstract

**Figure:**
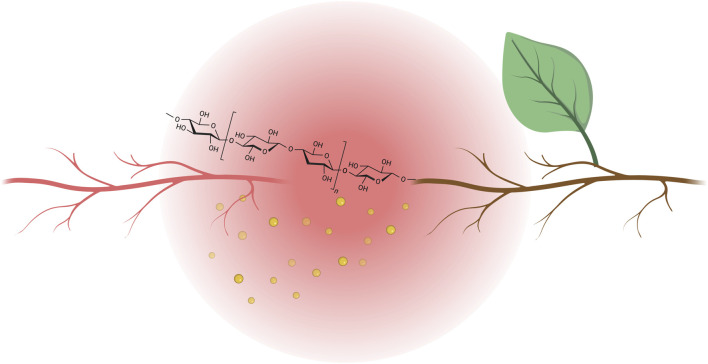

## 1 Introduction

Dense vascular networks ensure adequate oxygen and nutrient delivery and removal of metabolic waste from tissues (Carmeliet, 2000). Angiogenesis, the formation of new capillaries from pre-existing vessels, is upregulated in response to changes in the local environment such as trauma, ischemia, or tumorigenesis. It is triggered by release of angiogenic mediators including growth factors, enzymes, and reactive oxygen species (Meng et al., 2021). Gradients in these signals initiate endothelial cell activation and promote capillary sprouting, contributing to the structural organization of a new vascular network.

Angiogenesis is a complex process involving endothelial cell proliferation and migration, followed by lumen formation and stabilization through pericyte recruitment, smooth muscle cell coverage, and extracellular matrix (ECM) remodeling (Carmeliet, 2000; Calabriso et al., 2021). The tissue microenvironment plays a pivotal role in supporting angiogenic progression. However, the interplay between endothelial cells and the ECM is poorly understood, posing a barrier to research on drug screening, disease pathology and vascularized grafts for regenerative medicine (Meng et al., 2021; Libby et al., 2024). Improved understanding of the physiology and regulation of angiogenesis and vascularization has enabled the development of *in vitro* models, ranging from simple two-dimensional (2D) endothelial monocultures to advanced three-dimensional (3D) microfluidic systems, advancing vascular research (Huttala et al., 2015; Esparza et al., 2024; Yrjänäinen et al., 2024). There is increasing interest from regulatory bodies in developing new approach methodologies (NAMs) to reduce reliance on animal experiments in line with the 3Rs (Replacement, Reduction, Refinement) (Nitsche et al., 2025). At the same time, the development of xeno-free biomaterials enhances the potential of pre-vascularized systems to reach clinical applications.

Establishment of stable and functional microvascular networks *in vitro* remains a challenge. A key parameter is the distance between capillaries and cells, which cannot exceed 100–200 µm for sufficient nutrient and oxygen diffusion (Carmeliet and Jain, 2000). Although capillary density varies by tissue type, native capillaries are typically 1–8 µm in diameter and 1 mm in length and are usually separated by ∼40 µm (Murphy and Allenby, 2023). To recapitulate physiological vascularization, biomaterials must support the organization of endothelial cells into hierarchical networks and enable controlled presentation of angiogenic cues. Furthermore, the microenvironment must closely resemble native ECM to enable translation of *in vitro* findings. Hydrogels, typically composed of hydrophilic polymers in a hydrated 3D network, are interesting for this purpose due to their biocompatibility and capacity to support angiogenesis (Rosiak and Yoshii, 1999). Commonly used natural polymers, such as denatured collagen, hyaluronic acid, and elastin, are often derived from animals. Similarly, plant-derived polysaccharides e.g. alginate, are biocompatible but lack mechanical strength and stability, necessitating combination with synthetic materials or chemical cross-linkers. However, while synthetic polymers enhance mechanical properties, they can decrease biodegradability, biocompatibility, and tissue-specific modifiability. These limitations, coupled with ethical concerns regarding animal- or fossil-derived materials, highlight the need for standardized, biocompatible, and animal-free alternatives (Andrée et al., 2019).

Nanofibrillated cellulose hydrogel (NFCh) is a shear-thinning hydrogel derived from plants without chemical additives which has favorable properties for modelling angiogenesis, including tunable mechanical properties, physical mimicry of collagen fibrils, support for cell infiltration, hydrophilicity and batch consistency (Nitsche et al., 2025; Bhattacharya et al., 2012; Merivaara et al., 2022). These characteristics give NFCh potential for angiogenic research or therapies, where current biomaterials and protocols are unstandardized (Dai et al., 2014; Vailhé et al., 2001). In our previous study, we cultured human adipose-derived stromal cells (hASCs) and human umbilical vein endothelial cells (HUVECs) in NFCh and demonstrated 3D cell organization into capillary-like structures and differentiation into endothelial cells and pericytes demonstrating the potential of our model (Koivunotko et al., 2022). However, the study used monocultures and did not examine the effects of NFCh concentration and was not animal-free. Previous studies have examined the use of non-animal-based components in vascular research in 2D/3D and monocultures/cocultures (Esparza et al., 2024; Yrjänäinen et al., 2024; Andrée et al., 2019; Giles et al., 2025). However, the stiffness of these matrices cannot be easily tailored to match specific tissues as with NFCh. In this study several dilutions of NFCh are used to produce a 3D angiogenesis coculture model.

Building on our previous findings, we developed an animal component-free 3D *in vitro* angiogenesis model using HUVECs, hASCs in medium and human serum supplemented with medium and NFCh. First, the NFCh concentration was optimized in 3D HUVEC monocultures based on the amount of capillary-like organization. Then, cocultures of hASCs and HUVECs in NFCh were performed, the capillary-like structures were observed and compared to an animal-derived ECM control (Nitsche et al., 2025). In addition, the effects of NFCh stiffness on the angiogenic potential were examined by proteomics.

Although NFCh provides similar physical support as the physiological microenvironment, it does not reproduce specific protein interactions which support cell organization in natural ECM. This could be addressed in future studies, by combining human ECM compounds with NFCh. In addition, this model shows only a closed design of the vascularization lacking important environmental cues, such as the blood flow (Yrjänäinen et al., 2024). In future, NFCh could be included in organ-on-a-chip models.

Despite these limitations, we show for the first time the effects of varying NFCh concentration on cell organization and angiogenic potential without the use of animal-derived components. This study serves as a base for sustainable and tunable alternative materials for angiogenesis research. We hypothesized that NFCh could be used as an animal-free 3D *in vitro* platform for angiogenesis research and that fiber concentration could be tailored to modify cell behavior related to angiogenesis. The outcomes of this study may be translated into the development of standardized and reproducible *in vitro* models to reduce and replace the use of animals in research.

## 2 Materials and methods

### 2.1 Cells

HUVECs (PromoCell, Heidelberg, Germany, Cat. #C-12203, lot 488Z001) and hASCs (Lonza, Basel, Switzerland, Cat. #PT-5006, lot 22TL137983 and -137984) were used to create the angiogenesis model. HUVECs were thawed at passage 2 and subcultured every 4 days when approaching confluency by washing the cells with 1 × phosphate buffered saline without calcium or magnesium (DPBS) and then detaching with TrypLE (Thermo Fisher Scientific, MA, U.S.A) according to the manufacturer’s instructions. Cells were used between passages 3-4 and cultured in their growth medium EBM-2 Basal Medium (Cat. #CC-3156; Lonza) supplemented with 10% human serum (Cat. #H4522, Lot. 0000298193 and 2981889; Sigma-Aldrich).

For conditioned medium (CM) collection and coculture experiments, hASCs were thawed at passage 2 and subcultured every 4 days as described above and were cultured in their growth medium, MEM-α medium with GlutaMAX (MEM-α, Cat. #32571036; Gibco, UK) supplemented with 5% (v/v) human serum. Cells were used between passages 4-6 for collection of CM, and between passage 3-4 for coculture experiments. Cells were incubated at 37 °C in a humidified cell culture incubator with 5% CO_2_. The morphology and growth of the cells was tracked by brightfield microscopy (Leica DM IL LED microscope, Leica Microsystems, Wetzlar, Germany).

### 2.2 Three-dimensional cell culture matrix

Medical grade NFCh (FibGel, UPM Biomedicals, UPM-Kymmene) was used as a 3D matrix in cell culture experiments. NFCh stock (2.9% (m/w) fiber concentration) was diluted to final concentrations of 0.125%, 0.8%, 1.0%, 1.5%, or 2.4% with sterile ultrapure water to evaluate different stiffnesses in the model. The mechanical properties of each dilution was evaluated with rheology (Supplementary Material). Growth factor-reduced basement membrane extract of Engelbreth-Holm-Swarm mouse tumor (Matrigel, #356231, 0.2 g/mL, Merck, Germany) was diluted 1:6 with ice cold EBM-2 Basal medium as described previously (Mimler, 2025). After dilution, 50 µL/well was dispensed into 96-well plates (Sarstedt, Germany) and incubated for 30 min at 37 °C before the addition of cells.

### 2.3 Conditioned medium collection from 3D hASC culture for HUVEC stimulation

To produce CM, hASCs were grown to near confluency in 2D conditions and resuspended into a cell suspension of 1 × 10^6^ cells/mL in 0.125% NFCh and hASC growth medium. 100 µL/well of the suspension was dispensed into low-adhesion 96-well plates (inertGrade, BRAND plates, Cat. #BR781902; Merck, Darmstadt, Germany). After 20 min, 120 μL of growth medium was added on top of the NFCh. Medium was changed on days 1, 2, 3 and 5 by centrifuging the well plate (150 × g for 7 min) and exchanging the supernatant. During medium changes CM was collected and stored at −80 °C. Prior to use, CM was diluted 1:1 with EBM-2 Basal medium, henceforth, dCM.

### 2.4 Optimization of NFCh concentration for monoculture of HUVECs

**FIGURE 1:**
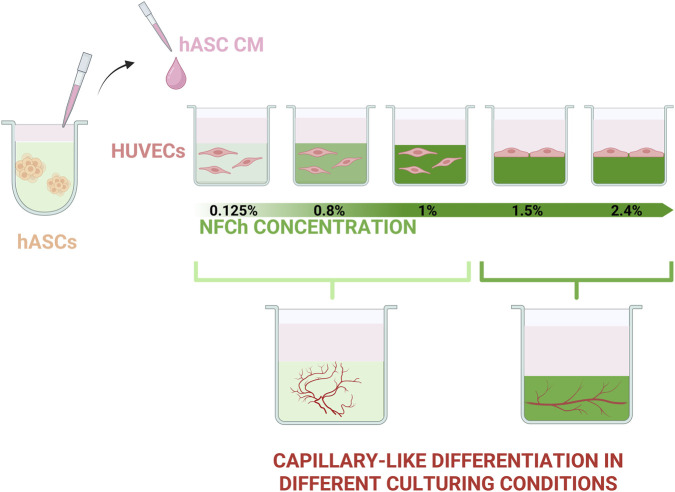
Illustration of human umbilical vein endothelial cells (HUVECs) monoculture in different nanofibrillated cellulose hydrogel (NFCh) concentrations (0.125%–2.4%). These systems were stimulated with conditioned medium (CM) from the three-dimensional (3D) human adipose-derived stromal cell (hASC) culture. Cultures without CM and two-dimensional (2D) cell culture were used as controls. The illustrative figure was created with Biorender.com.

The NFCh concentration for the coculture model was optimized based on the capillary-like organization in HUVEC monocultures. Figure 1 shows an illustration of the culture methods. Cells were grown to near confluence in 2D and detached with TrypLE after which cells were centrifuged at 210 x g for 5 min and the supernatant was aspirated. HUVECs were resuspended in HUVEC growth medium at a density of 6 × 10^5^ cells/mL and seeded with NFCh into 96-well plates to obtain 3D cultures as described previously (Koivunotko et al., 2022). Briefly, HUVECs were plated 50 µL/well in NFCh diluted between 0.125% and 1% with HUVEC growth medium. 3D cultured cells were incubated at 37 °C for 20 min after which 50 µL dCM was added on top. Since the stiffness of NFCh increases with concentration, HUVEC suspension (3 × 10^4^ per 50 µL) was overlaid on NFCh at concentrations of 1.5% and higher instead of resuspending. The medium was changed on days 3 and 5 as described before. 3D cultures without dCM and 2D cultures were used as controls. All monoculture systems had 4 parallel samples. After monoculture experiments, two NFCh concentrations with two different culture methods were chosen for coculture experiments with Matrigel as a coculture control.

### 2.5 Coculture of HUVECs and hASCs

**FIGURE 2:**
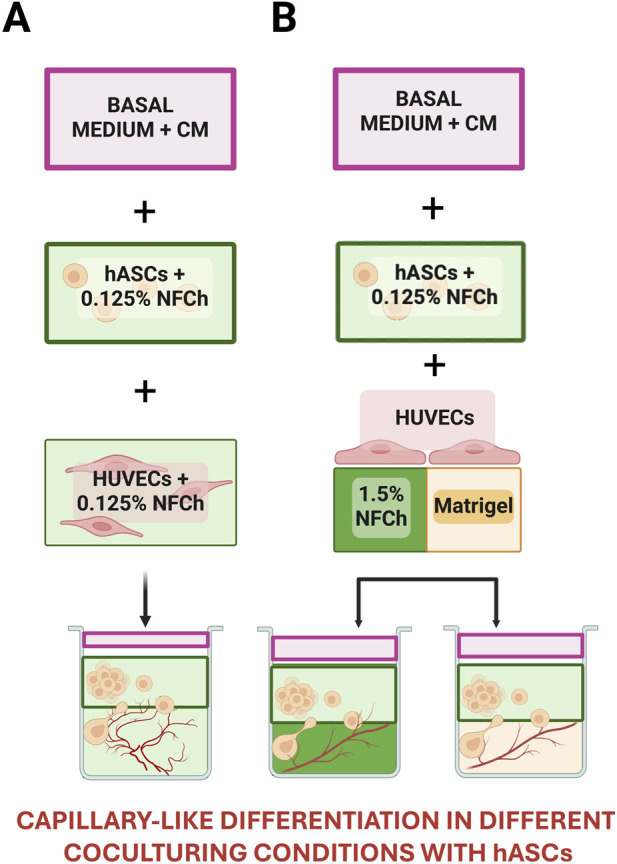
Illustration of the coculture systems. Coculturing with 0.125% nanofibrillated cellulose hydrogel (NFCh) using a cell-mixing technique **(A)**. Coculturing with 1.5% NFCh or Matrigel by culturing the cells on top of the matrix **(B)**. Human adipose-derivad stromal cells (hASCs) were mixed with 0.125% NFCh and cultured on top of the human umbilical vein endothelial cell (HUVEC) layer after centrifugation. The illustrative figure was created with Biorender.com.

Based on the measurements of the capillary-like structures in 3D HUVEC monocultures, (including vessel percentage area, total number of endpoints, average vessel length, total vessel length, total number of junctions and average vessel diameter), two NFCh concentrations (0.125%, 1.5%) with different culturing methods were chosen for separate 3D coculture experiments. Matrigel, diluted with medium 1:6, was used as animal-based material control. Figure 2 shows a schematic of the culture methods. The 3D coculture *in vitro* angiogenesis model contained HUVECs and hASCs using a sandwich culture technique. First, HUVECs were cultured in 3D for 3 days as previously described, medium was changed on day 2. On day 3, after centrifugation, the medium was removed and 50 µL of hASCs in 0.125% NFCh at a density of 6 × 10^5^ cells/mL were seeded on top of the culture system. After 10 min of incubation, 50 µL of dCM were added on top of the culture. The medium was changed on day 5. On day 7, cells were stained or collected for imaging and proteomic analyses. All the culture systems for the experiments were conducted with 4 parallel samples.

### 2.6 Immunocytochemistry of cell cultures

Both 3D monocultures and cocultures were stained for confocal microscopy as described elsewhere Koivunotko et al., (Koivunotko et al., 2022). The staining protocol was not animal-free and included bovine serum albumin and animal-derived antibodies but could be replaced with animal-free alternatives in future (Miri et al., 2026). Briefly, on day 7, both 3D monocultures and cocultures were fixed with cold 4% paraformaldehyde (PFA) with 0.8% Triton X-100, 5 mM EDTA (Cat. #E6758; Sigma-Aldrich), and 1 mM MgCl_2_ (Cat. #63063; Sigma-Aldrich) for 20 min at room temperature, after which the cells were washed three times with DPBS. Next, cells were blocked with 3% BSA and 0.3 M glycine in 1 × PBS with 0.5% (v/v) tween-20 (PBS-T) for 1 h at room temperature. Samples were incubated overnight at 4 °C with the primary antibodies (anti-rabbit CD31/PECAM (1:200; Cat. #NB100-2284), anti-rabbit vWF (1:200, #NBP1-84761) and anti-mouse NG2/MCSP (1:200, Cat. #NBP2-80873) all Novus Biologicals, Abingdon, UK) in 3% BSA in PBS-T. The next day, cells were washed three times with PBS-T and incubated with the appropriate secondary antibodies, (donkey anti-rabbit AlexaFluor594 1:500 (Cat. #A21207) and conjugated Phalloidin AlexaFluor488 (1:40, Cat. #A12379), both Thermo Fisher Scientific) in 5% BSA in PBS-T for 2 h at room temperature. Samples were washed three times with PBS-T and once with 0.1 M Tris-HCl pH 7.4 and incubated with 4 μg/mL Hoechst 33342 (Cat. #B2261; Sigma-Aldrich) in 0.1 M Tris-HCl for 2 h at room temperature. Samples were washed again with Tris-HCL and stored in PBS at +4 °C.

### 2.7 Semi-quantification of capillary-like structures

Samples were imaged with a Leica TCS SP8 STED 3X CW 3D confocal microscope (Leica microsystems). Images were taken at ×10 magnification and processed with Leica Application Suite X (Leica microsystems) and Fiji ImageJ 2.0.0-rc-69/1.52 n software (Schindelin et al., 2012). Between 1-4 independent images of 4 parallel samples per system were taken that were used for subsequent vessel quantifications. Additional Z-stack images constructed from 15 stack images with 33.8 µm total stack depth were taken to locate the cells in the whole 3D culture. The angiogenesis parameters, including vessel percentage area, total number of endpoints, average vessel length, total vessel length, total number of junctions and average vessel diameter, were semi-quantified using the Angiotool 2 plugin in Fiji (Supplementary Figure S1) (Zudaire et al., 2011). The analyses were semi-blinded as the images were re-labeled prior to quantification by the same researcher.

### 2.8 Proteomics of NFCh-based 3D cocultures

#### 2.8.1 Sample preparation

To evaluate changes in protein expression in the NFCh cocultures, LC/MS-MS proteomic analysis was performed on CM and cell lysates. CM was collected from 3D HUVEC cultures before the addition of hASCs on day 2 and from 3D cocultures on day 7. Cell lysates were collected on day 7 from the cocultures by degrading the NFCh with cellulase (GrowDase, UPM Biomedicals, UPM-Kymmene) overnight (37% v/v supplemented in HUVEC growth medium) according to the manufacturer’s instructions. Digested samples were centrifuged at 200 × g for 5 min, supernatant was removed, and pellets were washed with PBS after which 100 µL of EBM-2 Basal medium was added. Samples were stored at −80 °C until analysis.

#### 2.8.2 LC-MS/MS proteomics

LC-MS/MS proteomics were conducted in triplicate per system by the Viikki Proteomics Unit (University of Helsinki, Finland) as follows: 50 μg was taken from each sample and the volume was leveled to 200 μL with 100 mM AMBIC (Honeywell Fluka, cat. #15661030). The samples were reduced with 5 mM Tris(2-carboxyethyl)phosphine hydrochloride (#20490, Thermo Scientific), alkylated with 10 mM iodoacetamide (#122271000, Acros Organics) at room temperature. Reduced and alkylated peptides were trypsin-digested at 37 °C for 16 h using sequencing grade modified trypsin (V5113, Promega). After digestion, samples were acidified with 10% trifluoroacetic acid (Fisher, cat. #10723857) and desalted with BioPureSPN PROTO 300 C18 Mini columns (#HUM S18V, Higgins analytical, USA) according to manufacturer’s instructions. After desalting, the samples were dried in a centrifuge concentrator (Concentrator Plus, Eppendorf). The dried peptides were reconstituted in 30 µL buffer A (0.1% TFA, 1% acetonitrile (Fisher, cat. #10001334) in HPLC grade water (Fisher, cat. #10777404). Peptide concentration was measured with Pierce Quantitative Peptide Assays (Thermo Scientific, cat. #23275) and 150 ng of each sample was loaded into Evotips (Evosep) according to manufacturers’ instructions.

The samples were analyzed using the Evosep One liquid chromatography system coupled to a hybrid trapped ion mobility quadrupole TOF mass spectrometer (Bruker timsTOF Pro, Bruker Daltonics) (Meier et al., 2020) via a CaptiveSpray nano-electrospray ion source (Bruker Daltonics). An 8 cm × 150 µm column with 1.5 µm  C18 beads (EV1109, Evosep) was used for peptide separation with the 60 samples per day methods (21 min gradient time). Mobile phases A and B were 0.1% formic acid in water and 0.1% formic acid in acetonitrile, respectively. The MS analysis was performed in the positive-ion mode with dia-PASEF method (Meier et al., 2018) with sample optimized data independent analysis (dia) scan parameters. DDA was performed in PASEF mode from a pooled sample to be able to adjust dia-PASEF parameters optimally. To perform sample specific dia-PASEF parameter adjustment the default dia-short-gradient acquisition methods was adjusted based on the sample specific DDA-PASEF run with the software “tims Control” (Bruker Daltonics). The following parameters were modified for each sample type: m/z range: 425.0–1107.0 Da; mass steps per cycle: 29; mean cycle time: 1.17 s. The ion mobility windows were set to best match the ion cloud density from the sample type specific DDA-runs.

To analyze diaPASEF data, the raw data (.d) were processed with DIA-NN 2.2.0 Academia (Demichev et al., 2020; Demichev et al., 2022) using a spectral library generated from the UniProt human proteome (UP000005640, downloaded 06.02.2025 as a FASTA file, 20390 proteins). During library generation, the following settings were used, fixed modifications: carbamidomethyl (C); variable modifications: acetyl (protein N-term), oxidation (M); enzyme:Trypsin/P; maximun missed cleavages: 1; mass accuracy fixed to 1.5e-05 (MS2) and 1.5e-05 (MS1); Fragment m/z set to 200–1800; peptide length set to 7–50; precursor m/z set to 300–1800; Precursor changes set to 2–4; protein inference not performed. All other settings were left at default.

#### 2.8.3 Data analysis

A manually curated set of gene ontology biological process (GOBP) terms, selected based on domain relevance, was used to evaluate changes related to angiogenesis in cell lysates and CM, these were: GO:0060055, (*angiogenesis involved in wound healing,* 33 members), GO:1901343 (*negative regulation of vasculature development*, 167 members), GO:1904018 (*positive regulation of vasculature development,* 193 members), and GO:0048514 *(blood vessel morphogenesis,* 734 members). Similarly, stemness was evaluated based on the expression of general mesenchymal stem cell markers (THY1, NT5E, ENG), hASC phenotype protein markers present around blood vessels (PECAM1, MCAM), and GO:0072089 (*stem cell proliferation)* and GO:0097168 (*mesenchymal stem cell proliferation)*. The differentiation status of hASCs was analyzed based on the PanglaoDB pericyte marker dataset (Franzé et al., 2019).

For proteins not detected in all comparators, half-minimum value imputation was performed and then expression values were log2 transformed. Values present in both the experimental data and the chosen data sets were plotted as heatmaps with Euclidean hierarchical clustering using the ‘pheatmap’ and ‘viridis’ packages in R. In some cases, the fold change between relevant pairs of CM samples were calculated excluding proteins with only minimum-imputed values. The ‘Proteins with values/ranks” tool in StringDB was used to characterize these comparisons. The GOBP terms produced by StringDB were reduced by semantic similarity using the ‘rrvgo’ package in R. Treemaps were plotted using the ‘treemap’ package in R.

### 2.9 Statistical analyses

All quantitative data are presented as mean ± standard deviation (SD). For normally distributed data, statistical significance was determined using a one-way ANOVA with Tukey’s HSD *post hoc* test with IBM SPSS statistics (Version 27). The significance testing for vessel diameter was determined using a Kruskal–Wallis Dunn’s multiple comparison test. Significance was concluded when *p < 0.05, **p < 0.01 or ***p < 0.001.

## 3 Results

### 3.1 Optimization of NFCh concentration for HUVEC capillary-like organization

**FIGURE 3:**
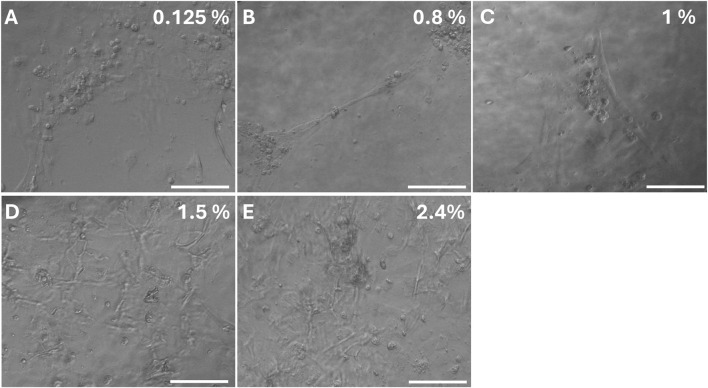
Brightfield micrographs of human umbilical vein endothelial cells (HUVECs) in 0.125% nanofibrillated cellulose hydrogel (NFCh) **(A)**, 0.8% NFCh **(B)**, 1% NFCh **(C)** 1.5% NFCh **(D)** and 2.4% NFCh **(E)** 3 days after seeding. Cells were organised into capillary-like morphology by day 3. Representative images have been taken with an inverted phase contrast microscope at ×10 magnification. Scale bar 200 µm. Percentages indicate NFCh concentration.

The NFCh concentration for the 3D angiogenesis model was determined by culturing HUVECs in 5 NFCh concentrations (0.125%, 0.8%, 1%, 1.5% and 2.4%) with increasing Young’s modulus (<0.5–2 kPa, Supplementary Figure S2). dCM-stimulated HUVECs formed spheroids by day 1 after seeding and capillary-like structures were observed on day 3 in every dilution (Figure 3). The densest capillary-like organization was estimated microscopically in 0.125% (Figure 3A), 1.5% (Figure 3D) and 2.4% (Figure 3E) NFCh concentrations. Organization in 0.8% was similar to 0.125% (Figure 3B). The presence of NFCh confounded microscopic detection of the cells in the 1% system, the highest concentration used with this method (Figure 3C). In systems without dCM, capillary-like morphology was not observed (Supplementary Figure S3), consistent with our previous results (Koivunotko et al., 2022).

**FIGURE 4:**
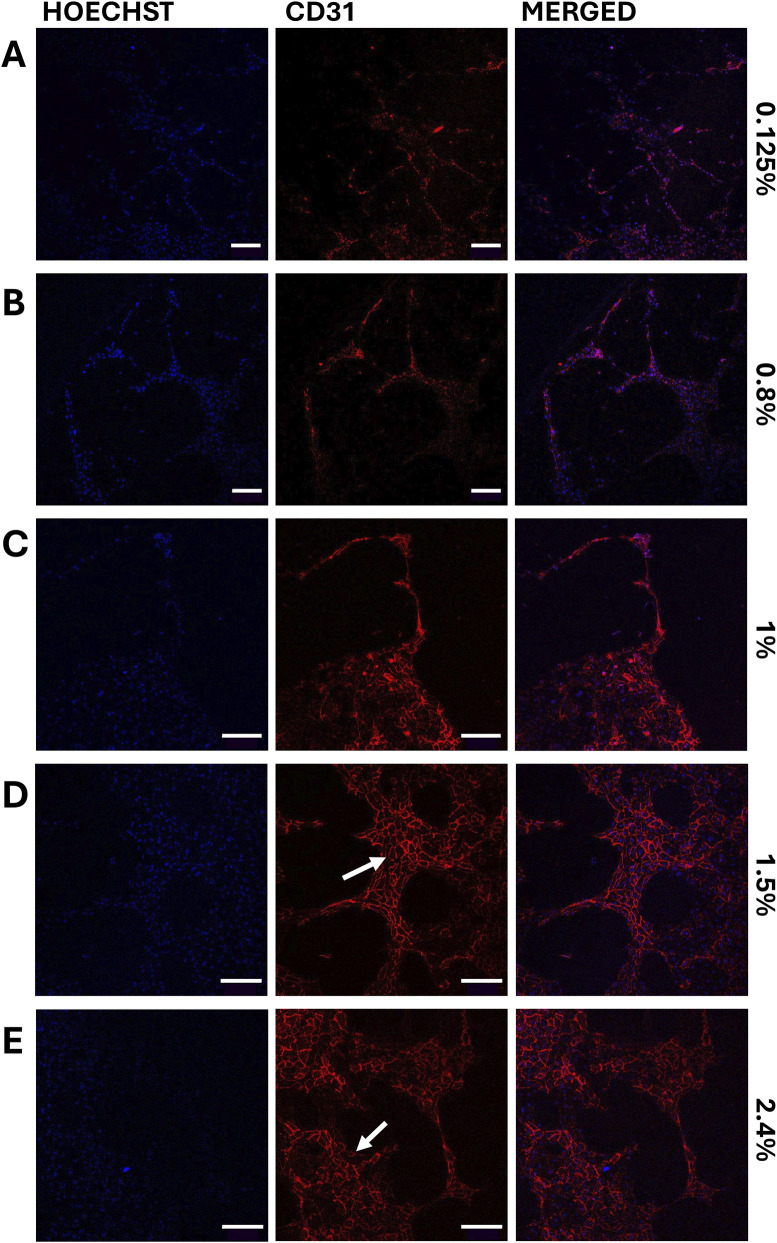
Single focal optical sections of CD31 stained human umbilical vein endothelial cells (HUVECs) to compare the endothelial cell morphological differences in different NFCh systems; 0.125% NFCh **(A)**, 0.8% NFCh **(B)**, 1% NFCh **(C)**, 1.5% NFCh **(D)** and 2.4% NFCh **(E)**. Three-dimensional (3D) cell cultures were fixed with PFA fixation solution without removing the hydrogel and stained with CD31 (red) and nuclei were stained with Hoechst (blue). Percentages indicate nanofibrillated cellulose hydrogel (NFCh) concentration. Capillary-like structures were observed in every system. White arrows indicate thicker capillary-like structures in 1.5% and 2.4% systems. Scale bar 200 µm.

In confocal microscopy, z-stacking was used to locate the cells, but analysis was conducted on single images, since most cells were observed in a single layer (Supplementary Figure S4). CD31 staining revealed similar structures to brightfield microscopy (Figure 4). Capillary-like structures were observed throughout the well surface area in 0.125%–0.8% NFCh systems (Figures 4A,B) and clustered to the well edges in 1.0%–2.4% (Figures 4C–E). In 1.5% and 2.4% NFCh, when cells were cultured on top of the matrix, cell density was increased and capillary-like structures covered large areas in the well (Figure 4, indicated as arrows).

**FIGURE 5:**
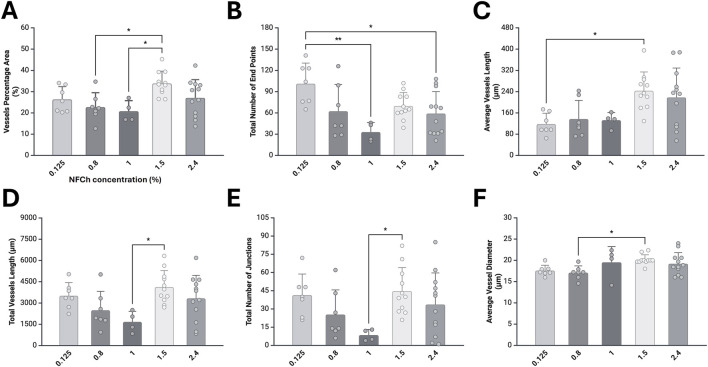
Vessel structure parameters in human umbilical vein endothelial cell (HUVEC) cultures in different nanofibrillated cellulose hydrogel (NFCh) concentrations. Each graph presents vessel percentage area **(A)**, total number of endpoints **(B)**, average vessel length **(C)**, total vessel length **(D)**, total number of junctions **(E)**, and average vessel diameter **(F)**. Grey bars denote the mean ± SD of 1-3 independent image of 4 parallel samples/system; hollow circles denote individual replicates. Significant differences are indicated by asterisks (*p < 0.05, **p < 0.01). Graphs were generated with Biorender.com and statistical analysis was carried out with IBM SPSS statistics software.

The capillary-like structures in the confocal micrographs were measured semi-quantitatively using Angiotool 2 in Fiji. The percentage area of the vessels (Figure 5A), number of endpoints (Figure 5B), mean length of vessel organization (Figures 5C,D), number of junctions in vessel structures (Figure 5E) and their diameter (Figure 5F) were calculated. As expected based on the poor cell detection in 1% NFCh, capillary-like structures in this condition had the lowest mean percentage area and shortest total vessel length with a low number of junctions. The highest structural differences in all parameters were observed in either 0.125% or 1.5% NFCh when compared with other systems. Therefore, these were chosen for subsequent coculture experiments in addition to their differences in culture methods. In 0.125% NFCh HUVECs were organized into the shortest vessel structures with the highest vessel number based on the number of end points and mean vessel length. No significant differences in vessel diameter were observed except in 0.8% where the vessels were narrower than in 1.5% (p = 0.03).

### 3.2 Comparable *in vitro* angiogenesis model between animal-free and animal-based coculture systems

**FIGURE 6:**
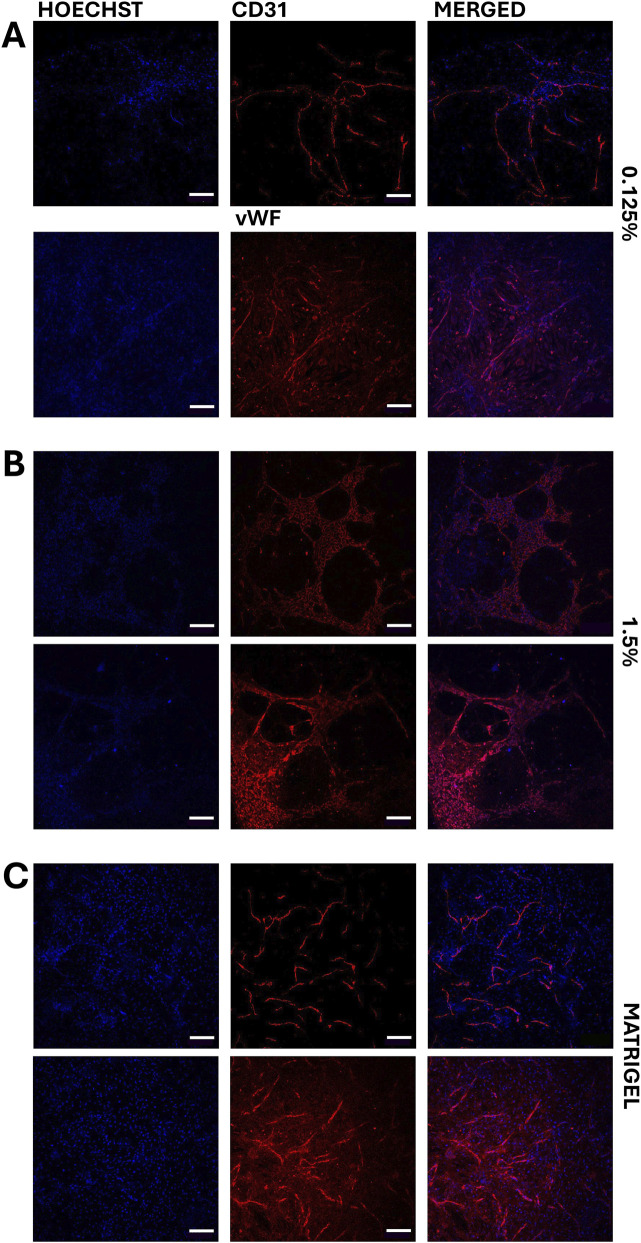
Single focal optical sections of CD31 or vWF stained cocultures of human umbilical vein endothelial cells (HUVECs) and human adipose-derived stromal cells (hASCs) to compare the morphological differences of the cell organizations in different hydrogel systems; 0.125% nanofibrillated cellulose hydrogel (NFCh) **(A)**, 1.5% NFCh **(B)** and Matrigel **(C)**. Three-dimensional (3D) cell cocultures were fixed with PFA fixation solution without removing the hydrogel and stained with CD31 or vWF (red) and nuclei were stained with Hoechst (blue). Percentages indicate NFCh concentration. Capillary-like structures were observed in every system. Scale bar 200 µm.

For cocultures, HUVECs were first stimulated in 0.125% or 1.5% NFCh or Matrigel with dCM for 3 days to form capillary-like structures and then cocultured with hASCs. Differentiation of hASCs into angiogenic cell types and changes in paracrine signaling affecting HUVEC organization was assessed. Based on microscopic evaluation on day 7, cocultures had higher cell density than monocultures and were organized into capillary-like structures, as expected. Cocultures were stained for endothelial cell markers CD31, vWF as well as early pericyte marker NG2 and imaged with confocal microscopy (Figures 6A–C). The fluorescence intensity of vWF was greater than CD31, but with higher standard deviation (Supplementary Figure S5). The smallest differences in CD31 and vWF intensity were in the 1.5% NFCh system. Conversely, CD31 staining revealed more organized capillary-like structures than vWF staining in every system. Visually, the Matrigel system had a similar capillary-like network to 0.125% NFCh, and 1.5% NFCh system had the highest stained cell density when visually compared to other systems. NG2 has been previously observed in NFCh-cultured hASCs (Koivunotko et al., 2022) but was not observed in the staining of this study suggesting absence of pericyte differentiation.

**FIGURE 7:**
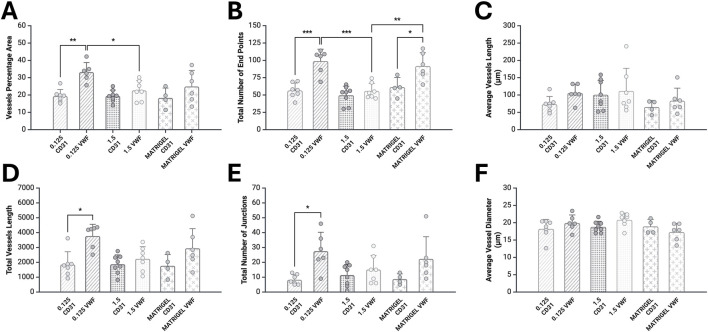
Semi-quantitative data of vessel structure parameters in human umbilical vein endothelial cell (HUVEC) and human adipose-derived stromal cell (hASC) cocultures stained with CD31 or vWF. Each graph presents vessel percentage area **(A)**, total number of endpoints **(B)**, average vessel length **(C)**, total vessel length **(D)**, total number of junctions **(E)**, and average vessel diameter **(F)**. Grey bars denote the mean ± SD of independent images; hollow circles denote 1-2 independent image of 4 parallel samples/system. Significant differences are indicated by asterisks *p < 0.05, **p < 0.01, ***p < 0.001.

Analysis of capillary-like structures showed the highest vessel percentage area (33%), highest number of end points (98.7) and junctions (27.5) and longest total vessel length (3769.1 um) in vWF stained 0.125% NFCh system that were significantly different to CD31 staining (19.4% p=0.002, 56.4 p < 0.001, 8.3 p = 0.02 and 1853.9 um p = 0.03, respectively). Similar significant differences were observed in the total number of endpoints in Matrigel system (vWF 91.7 versus CD31 61.3 p = 0.03). (Figures 7A–F). Conversely, 1.5% NFCh had the lowest vessel percentage area (22.7%) and the lowest number of end points (55.7) in vWF stained wells (p = 0.02 versus 0.125% NFCh, p < 0.001/p = 0.0011 versus 0.125% NFCh/Matrigel, respectively). In addition to stained cells, high vWF intensity may indicate released protein in the coculture systems (Mbagwu and Filgueira, 2020; Starke et al., 2011). Mean vessel diameter was similar in all systems and staining conditions (17.3 µm–20.8 µm).

### 3.3 Proteomic changes related to NFCh dilution in cells and CM

A total of 5,031 proteins were detected across all samples by global LC MS/MS proteomics. The samples were CM on days 2 (monoculture) and 7 (coculture) and cell pellets on day 7 (mono- and coculture). The majority of proteins were detected in cell pellets (mean 4,763.3 per sample) compared to a mean of 640.9 detected in CM samples.

**FIGURE 8:**
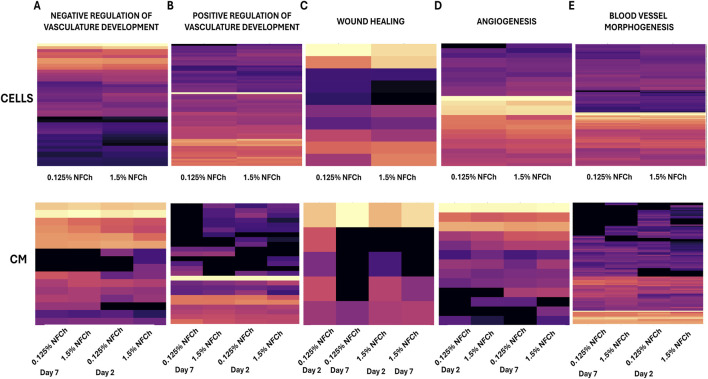
Heat maps of the log2 protein expression values of cell pellets and conditioned medium (CM). Different Gene Ontology Biological process (GOBP) datasets including negative regulation of vasculature development **(A)** positive regulation of vasculature development **(B)**, wound healing **(C)**, angiogenesis **(D)** and blood vessel morphogenesis **(E)** were used in the analyses. Brighter colours indicate higher expression. The dendrogram indicates Euclidean hierarchical clustering.

The changes in proteins expression related to vascularization are summarized as heat maps. The dataset of proteins related to angiogenesis were manually curated based on domain relevance. These were: vasculature development (Figures 8A,B), processes relating to vessel formation (angiogenesis and wound healing) (Figures 8C,D) and morphogenesis, (Figure 8E).

The expression profile of the tested samples was broadly similar. The greatest differences were observed temporally in CM samples of monoculture (day 2) and coculture (day 7). For instance, in both CM samples, ITGAV, THBS4 and CAMP were detected only on day 2 while ANGPTL4, MYDGF and IL6 were detected only on day 7. In addition, NFCh concentration was negatively correlated with protein expression related to wound healing in HUVEC monocultures, but positively correlated with protein expression related to vasculature development in cocultures compared with other CM systems. Differences in expression of the proangiogenic protein ANGPTL3 and the VEGF signaling regulator RHOB were observed between NFCh systems, since these proteins were detected only on day 2 and day 7 in 0.125% NFCh, respectively whereas the opposite pattern was observed in the 1.5% NFCh condition. Proteins regulating angiogenesis and vascularization related processes were expressed both in cell pellets and CM at each time point. Interestingly, VEGFA, which is a central signaling protein in angiogenesis was only detected in the 0.125% NFCh cell pellet (Lee et al., 2025).

**FIGURE 9:**
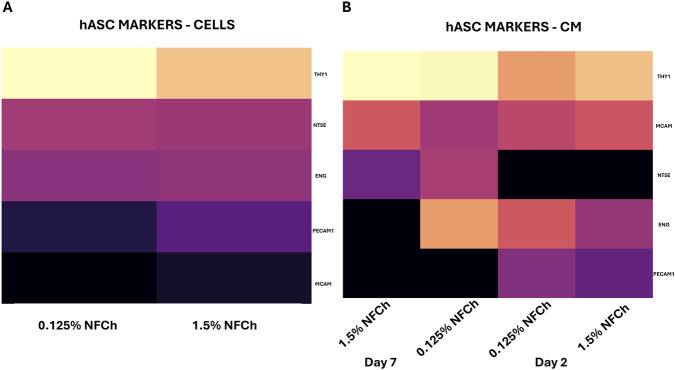
Heat maps of the log2 human adipose-derived stromal cells (hASC) stemness-related protein expression values found in cell pellets **(A)** and conditioned medium (CM) samples **(B)**. Protein intensities are showed as colours (yellow indicates the highest expression, black the lowest). Lines indicate the protein expression intensity in different culture systems.

Changes in hASC stemness, were evaluated based on expression of proteins in the GOBP terms “stem cell proliferation” and “mesenchymal stem cell proliferation” in cell pellets between day 2 and 7. The geometric mean of these fold-changes for both terms was not significantly different from 1 (p = 0.953 and p = 0.299, respectively) indicating no significant change in stemness over time in the culture. In addition, the expression of THY1, NT5E and ENG (hASC markers) and PECAM1, and MCAM (markers of stromal cells surrounding blood vessels) was visualized (Figure 9) (Saalbach and Anderegg, 2019; Baer, 2014). Each marker was expressed either in CM (Figure 9A) or cell samples (Figure 9B). Of these makers, the greatest changes were in NT5E and PECAM1 which were detected only on day 2 or day 7, respectively reflecting the changing composition of the culture.

**FIGURE 10:**
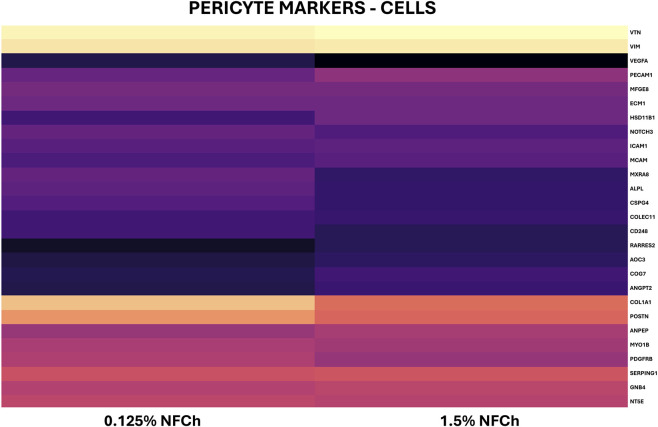
Heat maps of the log2 pericyte-related protein expression values found in cell pellets to evaluate the differentiation potential of human adipose-derived stromal cells (hASCs). Protein intensities are shown as colors (yellow indicates the highest expression, black the lowest). Lines indicate the protein expression intensity in different culture systems.

The differentiation status of cocultured hASCs was examined based on the expression of pericyte-associated proteins. Interestingly, although NG2 staining was not detected, NG2 protein expression was detected in both NFCh systems. The most highly expressed proteins in this dataset were VTN and VIM, that are characteristics of perivascular stromal cells and related to matrix remodeling (Figure 10). There were differences between NFCh conditions, in stiffer NFCh (1.5%), there was increased expression of ANGPT2, PECAM1, HSD11B1 and COG7. ANTGPT2 and PECAM1 are linked to vascular cell communication during angiogenesis. HSD11B1 and COG7 participate in metabolic regulation and matrix-driven remodeling, respectively. In contrast, MXRA8, and ALPL were more strongly expressed in the softer NFCh system, consistent with an immature mesenchymal or early perivascular phenotype and greater stromal plasticity.

The proteomics results were characterized with StringDB. Broadly, the changes in CM samples were associated with ECM remodeling which decreased from day 2 to day 7. On day 7, CM from 1.5% NFCh tended to show higher expression levels of these proteins. The processes associated with the proteomic changes in cell pellets were reduced by semantic similarity and visualized as a treemap (Supplementary Figure S6), which indicated nine major functional clusters. These analyses identified several biological processes relevant to angiogenesis and ECM dynamics. In softer NFCh (0.125%), enriched terms included wound-healing responses, ECM organization, paracrine signaling pathways, and factors linked to angiogenic activation (e.g., enrichment of ANGPTL3-associated processes). In contrast, stiffer NFCh (1.5%) collagen fibril organization, extracellular matrix structural assembly, and mechanotransduction-related processes were enriched, consistent with increased RHOB expression and higher expression of ECM-remodeling proteins.

## 4 Discussion

Angiogenesis was first demonstrated *in vitro* in the 1980s (Folkman and Haudenschild, 1980), initiating a rapidly expanding field which now encompasses complex *in vitro* models with promise for tissue regeneration applications. Angiogenesis research will be essential for engineering vascularized tissues and to understand pathological mechanisms underlying dysregulated vessel formation in diseases such as cancer (Kareva et al., 2016). However, the heterogeneity of angiogenesis in different physiological contexts, e.g. wound healing and tumor progression, makes standardizing experimental models and therapeutic strategies challenging. This issue is compounded by the widespread use of animal-derived matrices and serum-derived supplements, that suffer from batch variability, limited reproducibility, and ethical and xenogeneic concerns, motivating the search for animal-free alternatives for *in vitro* experiments (FDA, 2024; Meng and Day, 2026; Weber et al., 2025; Nguyen et al., 2017; Rosolowski et al., 2025).

Animal-free matrices for 3D angiogenesis models could increase the reproducibility of experiments and could even be translated into clinical applications. Researchers are developing alternative materials for 2D and 3D vascular models and expanding our understanding of commercially available alternatives such as the Basement Membrane Extract-free Database, demonstrating potential for future applications in compliance with 3Rs (Huttala et al., 2015; Andrée et al., 2019; 3Rs Centre Utrecht, 2025). Here, we demonstrated a proof-of-concept animal-free *in vitro* 3D angiogenesis model using a biocompatible plant-based matrix for vessel organization in human cells. We observed capillary-like structures formed by HUVECs with and without hASCs, noting both similarities and differences in cellular organization compared with a Matrigel-based model. These organizational features were modulated by adjusting the matrix stiffness, based on NFCh concentration. This capacity is an important advantage of NFCh for future applications.

The extracellular microenvironment is a critical regulator of angiogenesis, where both mechanical cues and cell–matrix interactions play integral roles in modulating endothelial cell behavior (Humphrey and Schwartz, 2021). *In vitro* studies have shown that substrate stiffness influences endothelial cell morphology, phenotype, proliferation, and organization, thereby shaping angiogenic potential under different physiological, or pathophysiological, conditions (Yeung et al., 2005; Xue et al., 2017; Wong et al., 2019). Although this study does not explicitly aim to replicate tissue-specific stiffness, the effects of NFCh fiber concentration on endothelial morphology suggest its suitability for tuning mechanical conditions in *in vitro* angiogenesis models.

There are contrasting reports on the effect of ECM stiffness on angiogenesis, with reports of enhanced sprouting in either stiffer or softer matrices (Bordeleau et al., 2017; Yeh et al., 2012; Leonard-Duke et al., 2023; Saunders and Hammer, 2010). These discrepancies likely arise from variations in material composition, stiffness ranges, and cell sources. Given concerns surrounding heterogeneous animal-derived matrices and synthetic materials with limited biocompatibility, the results herein support NFCh as a tunable and bioinert alternative for angiogenesis research. In the system described here, 2.4% NFCh had a stiffness of ∼2 kPa, 1.5% NFCh ∼0.5 kPa, and lower concentrations were below 0.5 kPa. Stiffer matrices supported higher and more unified vessel organization, which may also be related to differences in culture setup: embedding cells in softer matrices versus seeding cells on top of stiffer ones. In general, HUVECs self-organized into capillary-like networks across all monoculture NFCh conditions upon stimulation with dCM, demonstrating that NFCh supports angiogenic organization across a broad concentration range. Based on the monoculture results, two NFCh systems (0.125% and 1.5%), that differed in vessel parameters and culture techniques, were selected for 3D coculture studies. Sandwich-type coculture methods improve *in vitro* vascularization by increasing cell–matrix layering (Wu et al., 2022; Xu et al., 2023; Nishiguchi et al., 2014), and although this coculture setup used only 2 cell layers and two matrix layers, both NFCh concentrations supported successful vascular-like organization. In addition, the material composition of NFCh is water and sugar polymers without cross-linkers allowed its convenient removal with cellulase for analyses, ensuring non-interference.

Proteomic analysis showed minor differences in angiogenesis-related protein expression, supporting the applicability of multiple NFCh concentrations for angiogenesis models. For instance, PDGFRβ was expressed in both coculture systems, indicating that NFCh accommodates stromal–endothelial interactions crucial for angiogenesis. In addition, across all samples, C3, APOH, PLG, MYH9, and SERPINE1 were among the most abundant proteins, consistent with their established roles in angiogenesis, tissue repair, endothelial–stromal communication, and ECM remodeling in 3D coculture models (Wang et al., 2020; Lovett et al., 2010; Kachgal and Putnam, 2011).

Because angiogenesis is strongly governed by paracrine growth factors such as VEGF, PDGF, bFGF, and angiopoietins (Potente et al., 2011; Omorphos et al., 2021), and because dysregulated VEGF signaling is central in tumor angiogenesis and anti-angiogenic therapy (Liu et al., 2023), we evaluated total protein content in both CM and cell lysates from NFCh cocultures. Interestingly, VEGFA was detected only in cells cultured in 0.125% NFCh, consistent with downregulation of VEGFA under increasing stiffness (Santos et al., 2015). Other differences in proteomic analyses may have also related on stiffness-dependent phenotypes. For instance, ANGPTL3 was detected in 0.125% NFCh coculture but not in 1.5% NFCh, aligning with reports that ANGPTL3 expression decreases under mechanical stress (Zhu et al., 2023). Conversely, mechanosensitive proteins such as RHOB increased in the stiffer NFCh system. In 0.125% NFCh, proteins related to wound healing were elevated at day 2, while vasculature-related proteins were comparatively lower by day 7 than in 1.5% NFCh. Comparison of cell pellets from the two systems showed that softer NFCh promoted nuclear and transcriptional regulation, reflecting a more dynamic and paracrine-active phenotype. In contrast, higher stiffness supported biosynthetic and structural activity, consistent with cytoskeletal maintenance and ECM deposition that may also reflect the higher presence of vascular organization in stiffer NFCh cultures. Indeed, collagen cross-linking and matrix stiffening enhance ECM production in 3D cultured human mesenchymal stromal cells (Kersey et al., 2024). Overall, these findings show that NFCh provides a versatile, tunable platform for examining the mechanical regulation of angiogenesis while avoiding batch variation of animal-derived matrices.

Although coculturing endothelial and stromal cells typically enhances vascular-like organization (Rocha et al., 2020; Sarkanen et al., 2012; Cai et al., 2017), the cocultures in this study had lower vascular parameters than monocultures. This is likely attributable to experimental design factors rather than biological limitations of NFCh. Both culture types received potent pro-angiogenic stimulation from dCM, reducing the relative contribution of direct stromal–endothelial interactions. Additionally, hASCs were not precultured before contact with HUVECs, unlike earlier work demonstrating that pre-maturation enhances angiogenic sprouting (Song et al., 2016). Decreased vascularization could be attributed to reduced proliferation and viability of endothelial cells *in vitro* and *in vivo* when cocultured with mesenchymal stromal cells in synthetic hydrogel scaffolds (Pedersen et al., 2014). Together, these technical parameters including stiffness, timing, and layering strategy, are likely to shape the angiogenic output and should be refined in future experiments.

Despite some methodological concerns, clear evaluation of the staining distribution was made. Hoechst staining showed nuclei adjacent to vWF-positive structures, suggesting incomplete hASC differentiation. A similar spatial distribution has been reported in a 9-day 3D Matrigel cocultures (Terlizzi et al., 2018). Furthermore, the higher number of vWF-positive structures relative to CD31 staining in 0.125% NFCh and in Matrigel may reflect the strong stimulatory effect of hASCs on vWF expression (Follin et al., 2013) and aligns with previous reports that hASCs express higher vWF than CD31 under comparable conditions (Savkovic et al., 2021). In the 1.5% NFCh coculture, staining differences between vWF and CD31 were less pronounced, and nuclei appeared more closely aligned with endothelial-associated regions, possibly indicating more advanced differentiation and reduced vWF release. Based on these results, CD31 staining appears to be more effective for assessing angiogenic cell organization, whereas vWF may highlight the effects of hASCs better, though further optimization is required. It should be noted that staining used animal-derived antibodies, and future studies could use animal-free reagents to make the model fully animal-free. In addition, the staining protocol used Triton-X as a cell-permeabilizing agent. Due to its environmental harm, the European Economic Area has banned the compound with some exceptions for the pharmaceutical industry. In the future studies we aim to replace it with e.g. Tween-20 (Amidzadeh et al., 2014; Commission Regulation of the European Union, 2020).

The behavior of hASCs and their differentiation was characterized with proteomics. Both cell pellets and CM contained adipose stromal cell-associated markers, with expression patterns dependent on culture duration and matrix stiffness. As expected, NT5 expression increased with active hASC secretion, and MCAM was detected only in CM, potentially suggesting a soluble form with angiogenic relevance (Harhouri et al., 2010). Pericyte-associated proteins were studied to evaluate whether hASCs shifted toward a pericyte-like fate, as described previously where stromal cells were exposed to endothelial medium for 21 days (Koivunotko et al., 2022). While NG2/CSPG4 (Ozerdem and Stallcup, 2003) was not detected by confocal microscopy, 27 of 64 PanglaoDB pericyte markers were found in cell pellets. Interestingly NG2 was present in NFCh systems, which may indicate an early progenitor-like pericyte state and early angiogenic support functions. Despite partial expression of pericyte-related proteins, retention of stem-cell-associated signatures and the absence of key pericyte markers such as α-SMA indicate that differentiation was incomplete. The lack of mature supporting cells, which normally stabilize endothelial tubes and prevent excessive lumen enlargement, may also explain the larger vessel diameters observed here (18.2–20 µm) compared with native capillaries (2–8 µm) (Murphy and Allenby, 2023).

Current 3D vascular *in vitro* models span a broad spectrum of complexity, from traditional stromal–endothelial cocultures to microphysiological systems, organ-on-chip platforms, and stem-cell–derived vascular constructs (Yrjänäinen et al., 2024). While these systems have advanced our ability to recapitulate angiogenic processes, each comes with practical and biological limitations. Microfluidic devices can provide highly controlled gradients, interstitial flow, and compartmentalized architectures, yet many rely on closed-channel designs that restrict access, complicate cell layering, and limit nutrient and gas exchange. Even the most recent open-top devices capable of supporting two interacting 3D vascular networks require specialized fabrication, rely on fibrin or other biologically active matrices, and remain technically demanding to operate and reproduce. Stem-cell–derived vascular systems offer patient specificity and physiological relevance, but differentiation protocols are long, multi-step, and often require several media formulations with variable efficiency, making standardization difficult for routine angiogenesis studies (Esparza et al., 2024; Giles et al., 2025). Likewise, many NAMs and microphysiological models continue to depend on animal-derived ECM components, most commonly collagen or Matrigel, despite their ethical constraints, batch variability, and poorly defined bioactive composition, which complicates interpretation of angiogenic responses and reduces reproducibility across laboratories (Nitsche et al., 2025).

In contrast to these systems, the NFCh platform used in our study offers a fundamentally different type of experimental flexibility. Because NFCh is inert, it provides a matrix in which mechanical stiffness can be tuned by adjusting the concentration, without altering biochemical composition, which is difficult to achieve in protein-based hydrogels. This tunability aligns with the demand for standardizable, animal-free ECM alternatives highlighted across recent NAMs research. Particularly as reproducibility and transparency become key criteria for regulatory acceptance, animal-free alternatives could become a standardized method for *in vitro* research. Moreover, unlike complex animal-derived matrices which contain endogenous growth factors, NFCh allows exogenous control of biochemical cues, for instance by adding defined proteins, recombinant growth factors, or matrix-derived peptides. Such controlled augmentation parallels current trends in advanced vascular modeling, where synthetic or natural polysaccharide hydrogels are deliberately functionalized with adhesion motifs or growth factors to achieve targeted angiogenic behavior (Nitsche et al., 2025; Giles et al., 2025).

Although UPM Biomedicals, the manufacturer of NFCh in this study, withdrew from the market in 2025, NFCh has the potential to complement and integrate into the existing repertoire of 3D vascular *in vitro* model systems. Its tunable mechanics, bio-inert composition, and the compatibility with both mono- and coculture vascular organization, position it as an attractive material for next-generation angiogenesis platform, which highlights the potential for similar biomaterials to support the development of modular vascular assays adaptable to drug testing, disease modeling, and mechanobiology research.

## 5 Conclusion

This study demonstrates the use of an animal-free NFCh-based 3D angiogenesis model for research use. The model was comparable to widely used animal-derived 3D ECM system. Its stiffness can be tuned by adjusting NFCh concentration, without altering specific biological cues, or requiring temperature changes or cross-linkers. It is compatible with layered cocultures of angiogenic cell types. Overall, the results position NFCh as a valuable material for advancing angiogenesis modeling and for complementing established *in vitro* systems, with long-term potential to support translational biomaterial development.

## Data Availability

The original contributions presented in the study are publicly available. The mass spectrometry proteomics data have been deposited to the ProteomeXchange Consortium via the PRIDE partner repository with the dataset accession number PXD077881.

## Nomenclature

ALPL: Alkaline phosphatase
ANGPT2: Angiopoietin-2
ANGPTL-3: Angiopoietin-like 3
ANGPTL4: Angiopoietin-like 4
APOH: Apolipoprotein H (also known as β2-glycoprotein 1)
C3: Complement component 3
CAMP: Cathelicidin antimicrobial peptide
CM: Conditioned medium
COG7: Component of oligomeric Golgi complex 7
CSPG4/NG2: Chondroitin sulfate proteoglycan 4 (NG2)
dCM: Diluted conditioned medium
DPBS: Phosphate-buffered saline without calcium or magnesium
ECM: Extracellular matrix
ENG/CD105: Endoglin (CD105)
hASC(s): Human adipose-derived stromal cell(s)
HSD11B1: 11β-hydroxysteroid dehydrogenase type 1
HUVEC(s): Human umbilical vein endothelial cell(s)
GOBP: Gene Ontology Biological Process
ITGAV: Integrin alpha-V
MCAM/CD146: Melanoma cell adhesion molecule (CD146)
MXRA8: Matrix remodeling–associated protein 8
MYDGF: Myeloid-derived growth factor
MYH9: Myosin heavy chain 9
NAMs: New approach methodologies
NFCh: Nanofibrillated cellulose hydrogel
NT5E/CD73: 5′-nucleotidase ecto (CD73)
PBS-T: Phosphate-buffered saline with 0.5% (v/v) Tween-20
PDGFRβ: Platelet-derived growth factor receptor beta
PECAM1/CD31: Platelet/endothelial cell adhesion molecule 1 (CD31)
PLG: Plasminogen
RHOB: Ras homolog family member B
SD: Standard deviation
SERPINE1: Serpin family E member 1 (plasminogen activator inhibitor-1/PAI-1)
THBS4: Thrombospondin-4
THY1/CD90: Thy-1 cell surface antigen (CD90)
VEGFA: Vascular endothelial growth factor A
vWF: von Willebrand factor
2D: Two-dimensional
3D: Three-dimensional
3Rs: Replacement, reduction, refinement

## References

[B1] 3Rs Centre Utrecht (2025). Basement membrane extract (BME)-free database. RRIDSCR_026058. Available online at: https://bme-free.sites.uu.nl/(Accessed March 25, 2026).

[B2] Amidzadeh Z. Behbahani A. B. Erfani N. Sharifzadeh S. Ranjbaran R. Moezi L. (2014). Assessment of different permeabilization methods of minimizing damage to the adherent cells for detection of intracellular RNA by flow cytometry. Avicenna J. Med. Biotechnol. 6 (1), 38–46. PMID: 24523954; PMCID: PMC3895578. 24523954 PMC3895578

[B3] Andrée B. Ichanti H. Kalies S. Heisterkamp A. Strauß S. Vogt P. M. (2019). Formation of three-dimensional tubular endothelial cell networks under defined serum-free cell culture conditions in human collagen hydrogels. Sci. Rep. 9 (1), 5437. 10.1038/s41598-019-41985-6 30932006 PMC6443732

[B4] Baer P. C. (2014). Adipose-derived mesenchymal stromal/stem cells: an update on their phenotype *in vivo* and *in vitro* . World J. Stem Cells 6 (3), 256–265. 10.4252/wjsc.v6.i3.256 25126376 PMC4131268

[B5] Bhattacharya M. Malinen M. M. Lauren P. Lou Y. R. Kuisma S. W. Kanninen L. (2012). Nanofibrillar cellulose hydrogel promotes three-dimensional liver cell culture. J. Control. Release 164 (3), 291–298. 10.1016/j.jconrel.2012.06.039 22776290

[B6] Bordeleau F. Mason B. N. Lollis E. M. Mazzola M. Zanotelli M. R. Somasegar S. (2017). Matrix stiffening promotes a tumor vasculature phenotype. Proc. Natl. Acad. Sci. U. S. A. 114 (3), 492–497. 10.1073/pnas.1613855114 28034921 PMC5255592

[B7] Cai X. Xie J. Yao Y. Cun X. Lin S. Tian T. (2017). Angiogenesis in a 3D model containing adipose tissue stem cells and endothelial cells is mediated by canonical Wnt signaling. Bone Res. 5, 17048. 10.1038/boneres.2017.48 29263938 PMC5727463

[B8] Calabriso N. Stanca E. Rochira A. Damiano F. Giannotti L. di Chiara Stanca B. (2021). Angiogenic properties of concentrated growth factors (CGFs): the role of soluble factors and cellular components. Pharmaceutics 13 (5), 635. 10.3390/pharmaceutics13050635 33946931 PMC8146902

[B9] Carmeliet P. (2000). Mechanisms of angiogenesis and arteriogenesis. Nat. Med. 6 (4), 389–395. 10.1038/74651 10742145

[B10] Carmeliet P. Jain R. K. (2000). Angiogenesis in cancer and other diseases. Nature 407 (6801), 249–257. 10.1038/35025220 11001068

[B11] Commission Regulation of the European Union (2020). Commission Regulation (EU) 2020/2160 of 18 December 2020 Amending Annex XIV to Regulation (EC) No 1907/2006 of the European Parliament and of the Council L as Regards the Substance Group 4-(1,1,3,3-tetramethylbutyl)phenol, Ethoxylated (Covering well-defined Substances and Substances of Unknown or Variable Composition, Complex Reaction Products or Biological Materials, Polymers and Homologues). Luxembourg: Publication Office of the European Union, 431–438. Available online at: https://eur-lex.europa.eu/legal-content/EN/TXT/PDF/?uri=CELEX:32020R2160 (Accessed December 15, 2025).

[B12] Dai B. Zhang Y. Zhan Y. Zhang D. Wang N. He L. (2014). A novel tissue model for angiogenesis: evaluation of inhibitors or promoters in tissue level. Sci. Rep. 4, 3693. 10.1038/srep03693 24424154 PMC3892440

[B13] Demichev V. Messner C. B. Vernardis S. I. Lilley K. S. Ralser M. (2020). DIA-NN: neural networks and interference correction enable deep proteome coverage in high throughput. Nat. Methods 17 (1), 41–44. 10.1038/s41592-019-0638-x 31768060 PMC6949130

[B14] Demichev V. Szyrwiel L. Yu F. Teo G. C. Rosenberger G. Niewienda A. (2022). dia-PASEF data analysis using FragPipe and DIA-NN for deep proteomics of low sample amounts. Nat. Commun. 13 (1), 3944. 10.1038/s41467-022-31492-0 35803928 PMC9270362

[B15] Esparza A. Jimenez N. Borrego E. A. Browne S. Natividad-Diaz S. L. (2024). Review: human stem cell-based 3D *in vitro* angiogenesis models for preclinical drug screening applications. Mol. Biol. Rep. 51 (1), 260. 10.1007/s11033-023-09048-2 38302762 PMC10834608

[B16] Fda CBER (2024). Considerations for the use of human-and animal-derived materials in the manufacture of cellular and gene therapy and tissue-engineered medical products draft. Guid. Industry. Available online at: https://www.fda.gov/regulatory-information/search-fda-guidance-documents/considerations-use-human-and-animal-derived-materials-manufacture-cell-and-gene-therapy-and-tissue (Accessed December 15, 2025).

[B17] Folkman J. Haudenschild C. (1980). Angiogenesis *in vitro* . Nature 11 (288), 5791. 10.1038/288551a0 6160403

[B18] Follin B. Tratwal J. Haack-Sørensen M. Elberg J. J. Kastrup J. Ekblond A. (2013). Identical effects of VEGF and serum-deprivation on phenotype and function of adipose-derived stromal cells from healthy donors and patients with ischemic heart disease. J. Transl. Med. 11 (1), 219. 10.1186/1479-5876-11-219 24047149 PMC3852830

[B19] Franzén O. Gan L.-M. Björkegren J. L. M. (2019). PanglaoDB: a web server for exploration of mouse and human single-cell RNA sequencing data. Database 2019, baz046. 10.1093/database/baz046 30951143 PMC6450036

[B20] Giles R. Meijer E. M. Maas R. G. C. van Dijk C. G. M. Verhaar M. C. Cheng C. (2025). Animal-free alternatives for Matrigel in human iPSC-derived blood vessel organoid culture. Sci. Rep. 15 (1), 36042. 10.1038/s41598-025-20091-w 41094042 PMC12528733

[B21] Harhouri K. Kebir A. Guillet B. Foucault-Bertaud A. Voytenko S. Piercecchi-Marti M. D. (2010). Soluble CD146 displays angiogenic properties and promotes neovascularization in experimental hind-limb ischemia. Blood 115 (18), 3843–3851. 10.1182/blood-2009-06-229591 20185588

[B22] Humphrey J. D. Schwartz M. A. (2021). Vascular mechanobiology: homeostasis, adaptation, and disease. In Annu. Rev. Biomed. Eng. (23). 1, 27. 10.1146/annurev-bioeng-092419-060810 34255994 PMC8719655

[B23] Huttala O. Vuorenpää H. Toimela T. Uotila J. Kuokkanen H. Ylikomi T. (2015). Human vascular model with defined stimulation medium - a characterization study. Altex 32 (2), 125–136. 10.14573/altex.1411271 25742497

[B24] Kachgal S. Putnam A. J. (2011). Mesenchymal stem cells from adipose and bone marrow promote angiogenesis *via* distinct cytokine and protease expression mechanisms. Angiogenesis 14 (1), 47–59. 10.1007/s10456-010-9194-9 21104120 PMC3369878

[B25] Kareva I. Abou-Slaybi A. Dodd O. Dashevsky O. Klement G. L. (2016). Normal wound healing and tumor angiogenesis as a game of competitive inhibition. PLoS ONE 11 (12), e0166655. 10.1371/journal.pone.0166655 27935954 PMC5147849

[B26] Kersey A. L. Cheng D. Y. Deo K. A. Dubell C. R. Wang T. C. Jaiswal M. K. (2024). Stiffness assisted cell-matrix remodeling trigger 3D mechanotransduction regulatory programs. Biomaterials 306, 122473. 10.1016/j.biomaterials.2024.122473 38335719 PMC12861118

[B27] Koivunotko E. Snirvi J. Merivaara A. Harjumäki R. Rautiainen S. Kelloniemi M. (2022). Angiogenic potential of human adipose-derived mesenchymal stromal cells in nanofibrillated cellulose hydrogel. Biomedicines 10 (10), 2584. 10.3390/biomedicines10102584 36289846 PMC9599553

[B28] Lee C. Kim M. J. Kumar A. Lee H. W. Yang Y. Kim Y. (2025). Vascular endothelial growth factor signaling in health and disease: from molecular mechanisms to therapeutic perspectives. Signal Transduct. Target. Ther. 10 (1), 170. 10.1038/s41392-025-02249-0 40383803 PMC12086256

[B29] Leonard-Duke J. Bruce A. C. Peirce S. M. Taite L. J. (2023). Variations in mechanical stiffness alter microvascular sprouting and stability in a PEG hydrogel model of idiopathic pulmonary fibrosis. Microcirculation 30 (5–6), e12817. 10.1111/micc.12817 37248193 PMC10524245

[B30] Libby J. R. Royce H. Walker S. R. Li L. (2024). The role of extracellular matrix in angiogenesis: beyond adhesion and structure. In Biomater. Biosyst. 15. 100097. 10.1016/j.bbiosy.2024.100097 39129826 PMC11315062

[B31] Liu Z. L. Chen H. H. Zheng L. L. Sun L. P. Shi L. (2023). Angiogenic signaling pathways and anti-angiogenic therapy for cancer. Signal Transduct. Target. Ther. 8 (1), 198. 10.1038/s41392-023-01460-1 37169756 PMC10175505

[B32] Lovett M. Rockwood D. Baryshyan A. Kaplan D. L. (2010). Simple modular bioreactors for tissue engineering: a system for characterization of oxygen gradients, human mesenchymal stem cell differentiation, and prevascularization. Tissue Eng. - Part C. Methods 16 (6), 1565–1573. 10.1089/ten.tec.2010.0241 20528664 PMC2988631

[B33] Mbagwu S. I. Filgueira L. (2020). Differential expression of CD31 and von willebrand factor on endothelial cells in different regions of the human brain: potential implications for cerebral malaria pathogenesis. Brain Sci. 10 (1), 31. 10.3390/brainsci10010031 31935960 PMC7016814

[B34] Meier F. Brunner A.-D. Koch S. Koch H. Lubeck M. Krause M. (2018). Online parallel accumulation–serial fragmentation (PASEF) with a novel trapped ion mobility mass spectrometer. Mol. and Cell. Proteomics 17 (12), 2534–2545. 10.1074/mcp.TIR118.000900 30385480 PMC6283298

[B35] Meier F. Brunner A.-D. Frank M. Ha A. Bludau I. Voytik E. (2020). diaPASEF: parallel accumulation–serial fragmentation combined with data-independent acquisition. Nat. Methods 17 (12), 1229–1236. 10.1038/s41592-020-00998-0 33257825

[B36] Meng H. Day P. J. R. (2026). Exploring ethical, sustainable and effective foetal bovine serum alternatives for *in vitro* mammalian cell culture. Front. Toxicol. 8 (1776815), 1776815. 10.3389/ftox.2026.1776815 41878012 PMC13008496

[B37] Meng X. Xing Y. Li J. Deng C. Li Y. Ren X. (2021). Rebuilding the Vascular Network: *in vivo* and *in vitro* Approaches. Front. Cell Dev. Biol. 9, 639299. 10.3389/fcell.2021.639299 33968926 PMC8097043

[B38] Merivaara A. Koivunotko E. Manninen K. Kaseva T. Monola J. Salli E. (2022). Stiffness-Controlled hydrogels for 3D cell culture models. Polymers 14 (24), 5530. 10.3390/polym14245530 36559897 PMC9786583

[B39] Mimler T. (2025). Smooth muscle cells and endothelial cells: comparative Study of 3D culture in GrowDex and matrigel™. Ap-Plication Note 32. Available online at: www.upmbiomedicals.com/resource-center/applicationnotes/smooth-muscle-cells-and-endothelial-cells-comparative-study-of-3dculture-ingrowdex-matrigel/(Accessed December 15 2025).

[B40] Miri Z. Laakkonen J. Toivonen E. Väljä N. Miettinen S. Vuorenpää H. (2026). Establishment of human-relevant *in vitro* models using animal-free serum replacement and recombinant antibodies. Front. Toxicol. 8 (1741716), 1741716. 10.3389/ftox.2026.1741716 41938738 PMC13047193

[B41] Murphy A. R. Allenby M. C. (2023). *In vitro* microvascular engineering approaches and strategies for interstitial tissue integration. Acta Biomater. 171, 114–130. 10.1016/j.actbio.2023.09.019 37717711

[B42] Nguyen E. H. Daly W. T. Le N. N. T. Farnoodian M. Belair D. G. Schwartz M. P. (2017). Versatile synthetic alternatives to Matrigel for vascular toxicity screening and stem cell expansion. Nat. Biomed. Eng. 1 (7). 10.1038/s41551-017-0096 29104816 PMC5667681

[B43] Nishiguchi A. Matsusaki M. Asano Y. Shimoda H. Akashi M. (2014). Effects of angiogenic factors and 3D-microenvironments on vascularization within sandwich cultures. Biomaterials 35 (17), 4739–4748. 10.1016/j.biomaterials.2014.01.079 24655783

[B44] Nitsche K. S. Carmichael P. L. Malcomber S. Müller I. Bouwmeester H. (2025). Alternatives to animal-derived extracellular matrix hydrogels? An explorative study with HepaRG cells in animal-free hydrogels under static and dynamic culture conditions. Front. Toxicol. 7, 1649393. 10.3389/ftox.2025.1649393 41244631 PMC12611971

[B45] Omorphos N. P. Gao C. Tan S. S. Sangha M. S. (2021). Understanding angiogenesis and the role of angiogenic growth factors in the vascularisation of engineered tissues. Mol. Biol. Rep. 48 (1), 941–950. 10.1007/s11033-020-06108-9 33393005

[B46] Ozerdem U. Stallcup W. B. (2003). Early contribution of pericytes to angiogenic sprouting and tube formation. Angiogenesis 6 (3), 241–249. 10.1023/B:AGEN.0000021401.58039.a9 15041800 PMC1371062

[B47] Pedersen T. O. Blois A. L. Xue Y. Xing Z. Sun Y. Finne-Wistrand A. (2014). Mesenchymal stem cells induce endothelial cell quiescence and promote capillary formation. Stem Cell Res. Ther. 5 (1), 23. 10.1186/scrt412 24533904 PMC4055064

[B48] Potente M. Gerhardt H. Carmeliet P. (2011). Basic and therapeutic aspects of angiogenesis. Cell 146 (6), 873–887. 10.1016/j.cell.2011.08.039 21925313

[B49] Rocha L. A. Gomes E. D. Afonso J. L. Granja S. Baltazar F. Silva N. A. (2020). *In vitro* evaluation of ASCs and HUVECs cocultures in 3D biodegradable hydrogels on neurite outgrowth and vascular Organization. Front. Cell Dev. Biol. 8, 489. 10.3389/fcell.2020.00489 32612997 PMC7308435

[B50] Rosiak J. M. Yoshii F. (1999). Hydrogels and their medical applications. Nucl. Instrum. Methods Phys. Res. Sect. B Beam Interact. Mater. Atoms 151 (1–4), 56–64. 10.1016/S0168-583X(99)00118-4

[B51] Rosolowski J. Weber T. Malakpour-Permlid A. Oredsson S. (2025). Revisiting 3Rs: rethinking replacement and new approach methodologies. Front. Toxicol. 7 (7), 1664209. 10.3389/ftox.2025.1664209 41322701 PMC12660069

[B52] Saalbach A. Anderegg U. (2019). Thy-1: more than a marker for mesenchymal stromal cells. FASEB J. 33 (6), 6689–6696. 10.1096/fj.201802224R 30811954

[B53] Santos L. Fuhrmann G. Juenet M. Amdursky N. Horejs C. M. Campagnolo P. (2015). Extracellular stiffness modulates the expression of functional proteins and growth factors in endothelial cells. Adv. Healthc. Mater. 4 (14), 2056–2063. 10.1002/adhm.201500338 26270789

[B54] Sarkanen J. R. Vuorenpää H. Huttala O. Mannerstrm B. Kuokkanen H. Miettinen S. (2012). Adipose stromal cell tubule network model provides a versatile tool for vascular research and tissue engineering. Cells Tissues Organs 196 (5), 385–397. 10.1159/000336679 22739504

[B55] Saunders R. L. Hammer D. A. (2010). Assembly of human umbilical vein endothelial cells on compliant hydrogels. Cell. Mol. Bioeng. 3 (1), 60–67. 10.1007/s12195-010-0112-4 21754971 PMC3132815

[B56] Savkovic V. Li H. Obradovic D. Masieri F. F. Bartella A. K. Zimmerer R. (2021). The angiogenic potential of mesenchymal stem cells from the hair follicle outer root sheath. J. Clin. Med. 10 (5), 911. 10.3390/jcm10050911 33652691 PMC7956349

[B57] Schindelin J. Arganda-Carreras I. Frise E. Kaynig V. Longair M. Pietzsch T. (2012). Fiji: an open-source platform for biological-image analysis. Nat. Methods 9 (7), 676–682. 10.1038/nmeth.2019 22743772 PMC3855844

[B58] Song Y. H. Shon S. H. Shan M. Stroock A. D. Fischbach C. (2016). Adipose-derived stem cells increase angiogenesis through matrix metalloproteinase-dependent collagen remodeling. Integr. Biol. (United Kingdom) 8 (2), 205–215. 10.1039/c5ib00277j 26758423 PMC4755818

[B59] Starke R. D. Ferraro F. Paschalaki K. E. Dryden N. H. McKinnon T. A. J. Sutton R. E. (2011). Endothelial von Willebrand factor regulates angiogenesis. Blood 117 (3), 1071–1080. 10.1182/blood-2010-01-264507 21048155 PMC3035068

[B60] Terlizzi V. Kolibabka M. Burgess J. K. Hammes H. P. Harmsen M. C. (2018). The pericytic phenotype of adipose tissue-derived stromal cells is promoted by NOTCH2. Stem Cells 36 (2), 240–251. 10.1002/stem.2726 29067740

[B61] Vailhé B. Vittet D. Feige J. J. (2001). *In vitro* models of vasculogenesis and angiogenesis. Lab. Investig. 81 (4), 439–452. 10.1038/labinvest.3780252 11304563

[B62] Wang X. Zhang M. Ma J. Xu M. Chang J. Gelinsky M. (2020). 3D printing of cell-container-like scaffolds for Multicell Tissue engineering. Engineering 6 (11), 1276–1284. 10.1016/j.eng.2020.08.001

[B63] Weber T. Malakpour-Permlid A. Chary A. D’Alessandro V. Haut L. Seufert S. (2025). Fetal bovine serum: how to leave it behind in the pursuit of more reliable science. Front. Toxicol. 7 (1612903), 1612903. 10.3389/ftox.2025.1612903 40861932 PMC12371577

[B64] Wong L. Kumar A. Gabela-Zuniga B. Chua J. Singh G. Happe C. L. (2019). Substrate stiffness directs diverging vascular fates. Acta Biomater. 96, 321–329. 10.1016/j.actbio.2019.07.030 31326665

[B65] Wu M. Chen F. Liu H. Wu P. Yang Z. Zhang Z. (2022). Bioinspired sandwich-like hybrid surface functionalized scaffold capable of regulating osteogenesis, angiogenesis, and osteoclastogenesis for robust bone regeneration. Mater. Today Bio 17, 100458. 10.1016/j.mtbio.2022.100458 36278143 PMC9583582

[B66] Xu J. Zhang L. Ye Z. Chang B. Tu Z. Du X. (2023). A 3D “sandwich” coculture system with vascular niche supports mouse embryo development from E3.5 to E7.5 *in vitro* . Stem Cell Res. Ther. 14 (1), 349. 10.1186/s13287-023-03583-2 38072932 PMC10712047

[B67] Xue C. Zhang T. Xie X. Zhang Q. Zhang S. Zhu B. (2017). Substrate stiffness regulates arterial-venous differentiation of endothelial progenitor cells *via* the Ras/Mek pathway. Biochimica Biophysica Acta - Mol. Cell Res. 1864 (10), 1799–1808. 10.1016/j.bbamcr.2017.07.006 28732675

[B68] Yeh Y. T. Hur S. S. Chang J. Wang K. C. Chiu J. J. Li Y. S. (2012). Matrix stiffness regulates endothelial cell proliferation through Septin 9. PLoS ONE 7 (10), e46889. 10.1371/journal.pone.0046889 23118862 PMC3485289

[B69] Yeung T. Georges P. C. Flanagan L. A. Marg B. Ortiz M. Funaki M. (2005). Effects of substrate stiffness on cell morphology, cytoskeletal structure, and adhesion. Cell Motil. Cytoskelet. 60 (1), 24–34. 10.1002/cm.20041 15573414

[B70] Yrjänäinen A. Mesiä E. Lampela E. Kreutzer J. Vihinen J. Tornberg K. (2024). Barrier-free, open-top microfluidic chip for generating two distinct, interconnected 3D microvascular networks. Sci. Rep. 14 (1), 22916. 10.1038/s41598-024-74493-3 39358415 PMC11447027

[B71] Zhu M. Wang Q. Gu T. Han Y. Zeng X. Li J. (2023). Hydrogel-based microenvironment engineering of haematopoietic stem cells. Cell. Mol. Life Sci. 80 (2), 49. 10.1007/s00018-023-04696-w 36690903 PMC11073069

[B72] Zudaire E. Gambardella L. Kurcz C. Vermeren S. (2011). A computational tool for quantitative analysis of vascular networks. PLoS ONE 6 (11), e27385. 10.1371/journal.pone.0027385 22110636 PMC3217985

