# Supplementary figures and images for: Animal-free matrix alternative for three-dimensional in vitro angiogenesis models
Source: Front Toxicol. 2026 Jun 18;8:1768268. doi: 10.3389/ftox.2026.1768268 (PMC13322768; doi:10.3389/ftox.2026.1768268)

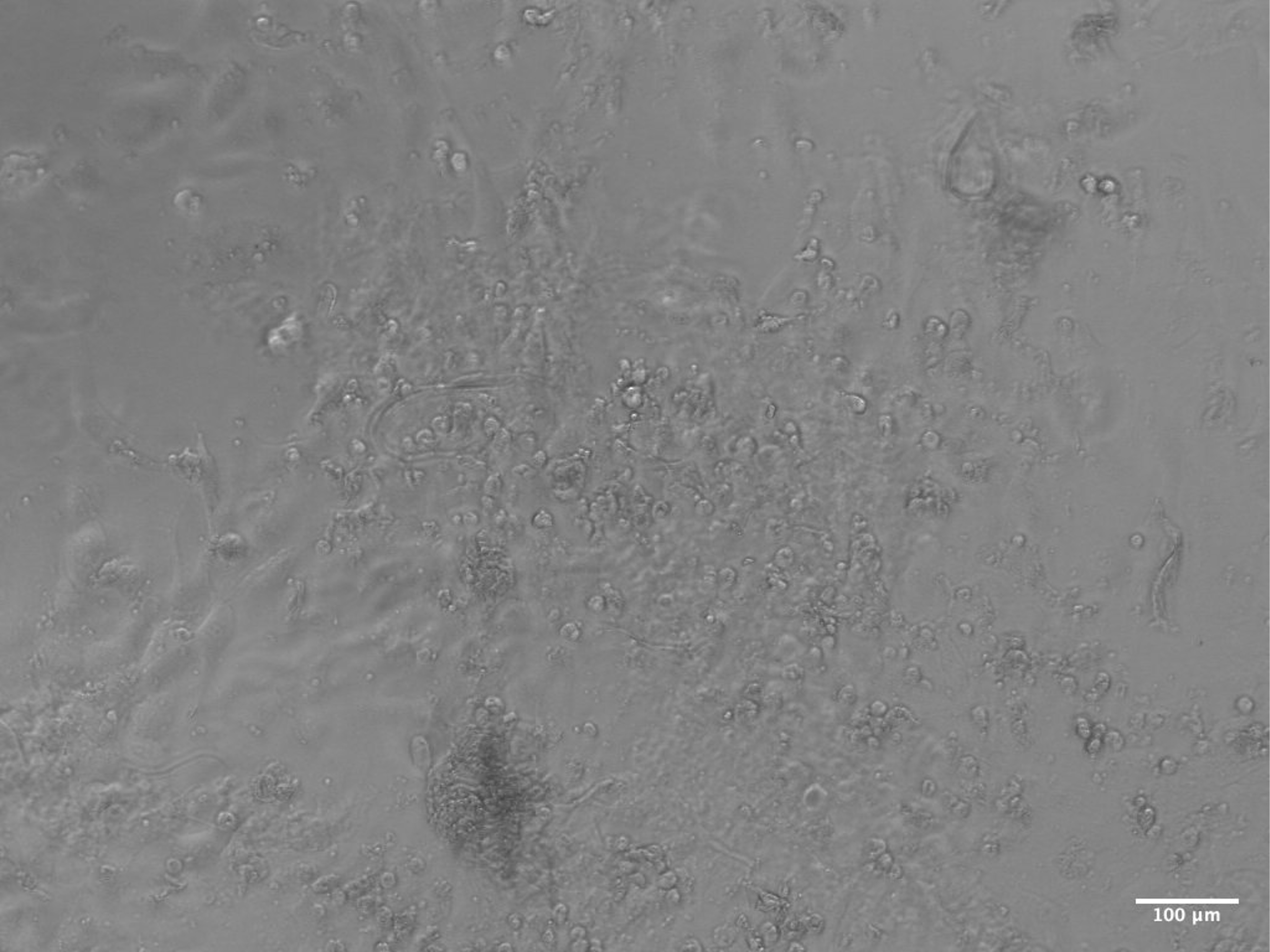

Supplement: Supplementary file 1 [file Image3.jpeg]

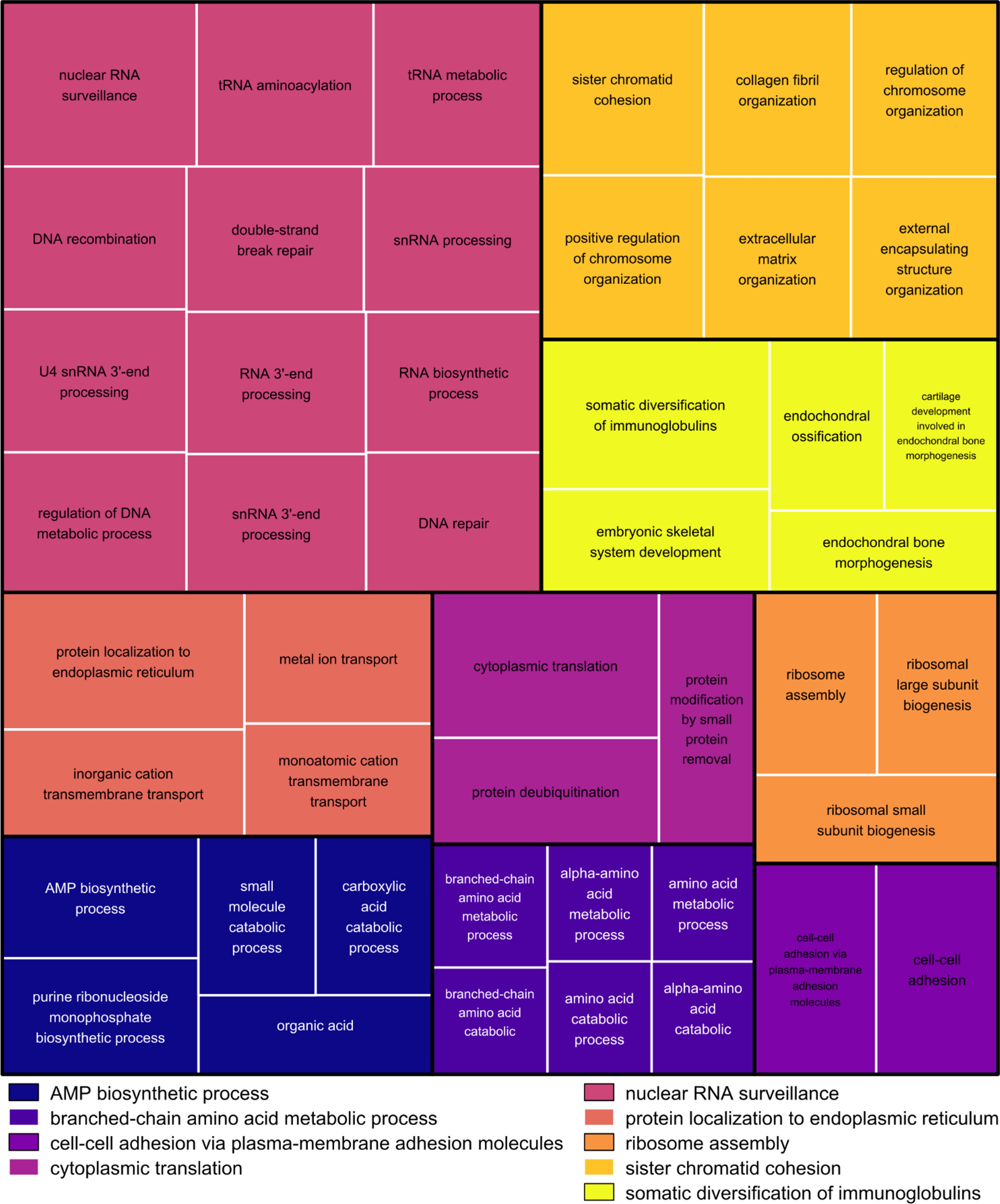

Supplement: Supplementary file 7 [file Image6.jpeg]
