# Supplementary material for: Animal-free matrix alternative for three-dimensional in vitro angiogenesis models
Source: Front Toxicol. 2026 Jun 18;8:1768268. doi: 10.3389/ftox.2026.1768268 (PMC13322768; doi:10.3389/ftox.2026.1768268)
Supplement: Supplementary file 2 [file Supplementaryfile1.docx]

Supplementary Material

# ANIMAL-FREE MATRIX ALTERNATIVE FOR THREE-DIMENSIONAL *IN VITRO* ANGIOGENESIS MODEL

*Elle Koivunotko^1^, Chris S. Pridgeon^1^, Lauri Paasonen^2^, Riina Harjumäki^1^*

*^1^Division of Pharmaceutical Biosciences, Drug Research Program, Faculty of Pharmacy, University of Helsinki, 00790 Helsinki, Finland; ^2^UPM Biomedicals, UPM-Kymmene Corporation, 00100 Helsinki, Finland.*

## Materials and methods

### 1.1 Rheological measurements of nanofibrillated cellulose hydrogel dilutions

Mechanical stiffness of each nanofibrillated cellulose hydrogel (NFCh) dilution was based on Young’s modulus measurements. The rheological measurements were made following the protocol described previously (1) using a HAAKE Viscotester iQ Rheometer (Thermo Fisher Scientific, Karlsruhe, Germany). Briefly, shear loss modulus (G ″) and shear storage modulus (G ′) were measured at 25 °C using cone plate geometry with a 0.1 mm gap for 1.0-2.9% NFC hydrogel dilutions and double cap geometry with a 4 mm gap for 0.125% and 0.8% dilutions. The oscillatory stresses based on the linear regions were τ =0.5 Pa (0.125%), 2 Pa (0.8%), 5 Pa (1 and 1.5%) or 7 Pa (3%) with an angular frequency range of 1-9 rads^-1^. Young`s modulus was calculated at frequency point 4.9 rads^-1^ as follows:

$$\boldsymbol{\surd}{\boldsymbol{(G}\boldsymbol{'}}^{\boldsymbol{2}}\boldsymbol{+}{\boldsymbol{G}\boldsymbol{''}}^{\boldsymbol{2}}\boldsymbol{)}$$

### 1.2 Semi-quantification of vessel-like structures

All confocal images of monoculture and coculture systems were semi-quantitatively analysed using AngioTool 2 (2). Images from both CD31 stained cells and vWF stained cells were used in the analysis. The following angiogenesis-related parameters were obtained: vessel percentage, vessel length and diameter and number of end points. In figure S1 an example of the AngioTool 2 analyses and equivalent CD31 stained cells in 0.125% NFC hydrogel coculture are presented.

*
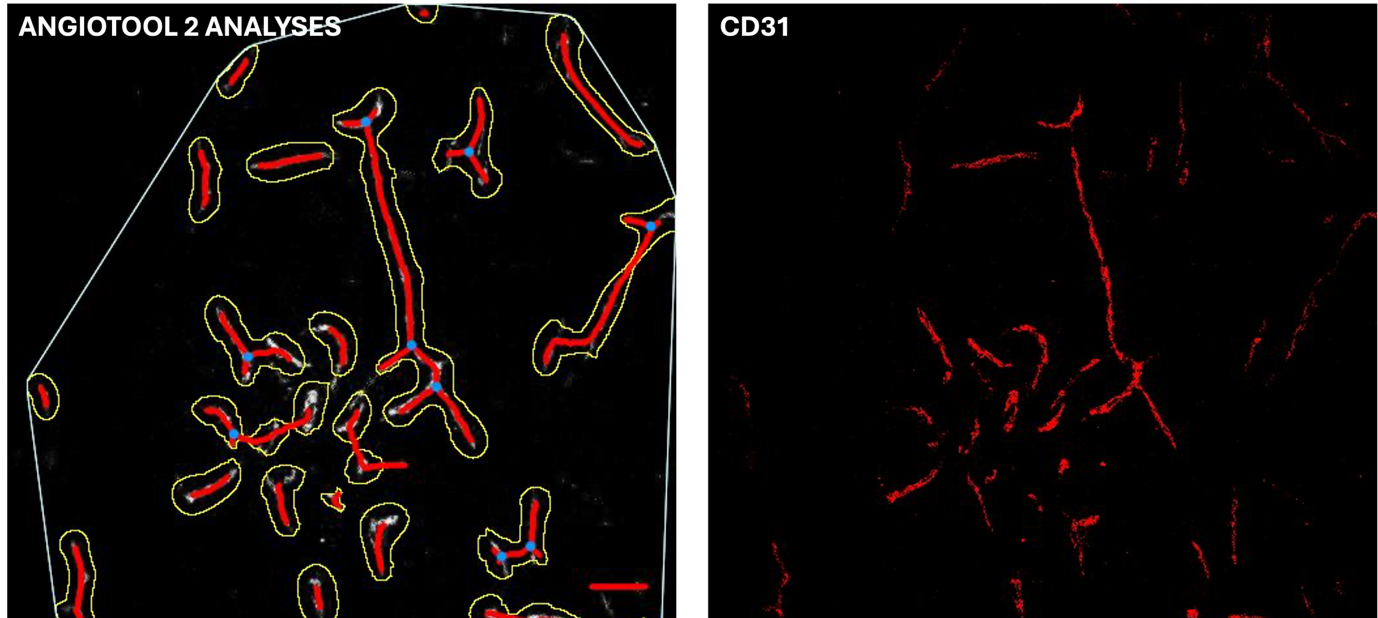
*

**Figure S1**. Example of AngioTool 2 analysis. The leftmost image shows vessel analysis in which red lines indicate detected blood vessels, blue indicates junction points and yellow the outline of the vessel. The rightmost image is the original image of the 0.125% coculture system used for angiogenesis analysis.

## Results

### 2.1 Increased mechanical stiffness with increasing NFCh concentration

G ′ and G ″ measurements from different NFCh concentrations were used to calculate Young`s Moduli (Figure 2). As expected, with higher fiber concentration higher mechanical stiffness was obtained.

**
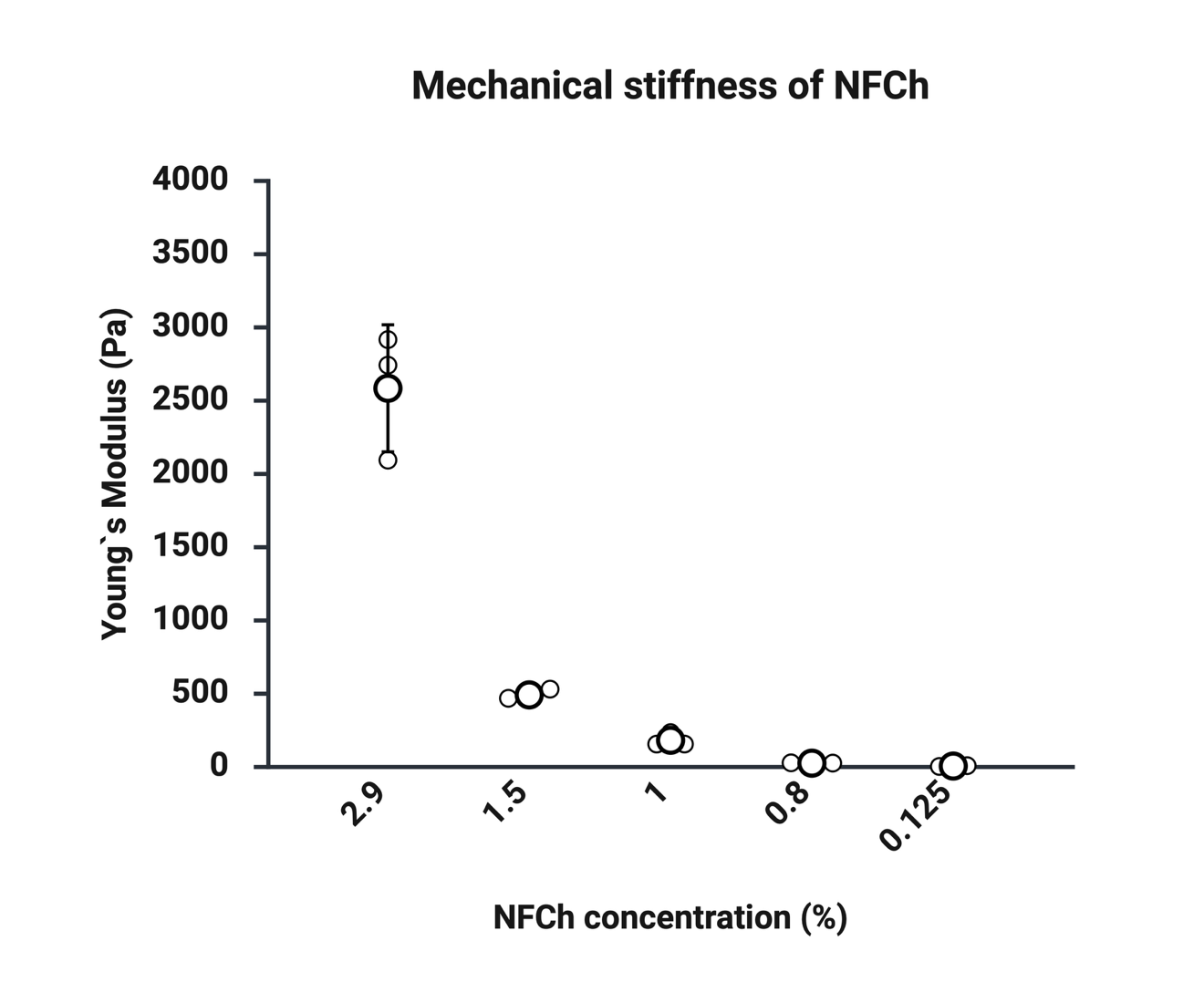
Figure S2.** Calculated Young`s moduli from different nanofibrillated cellulose hydrogel (NFCh) dilutions.

### 2.2 HUVECs formed spheroids in 3D cultures without CM stimulation

As in our previous study (3), human umbilical vein endothelial cells (HUVECs) without human adipose-derived stromal cell (hASC)-derived conditioned media (CM) stimulation did not organize into capillary-like structures. Cells were distributed evenly in the culture system and formed spheroids. An example of the HUVEC organization without CM stimulation in 0.125% on day 4 is presented in Figure S3.

**
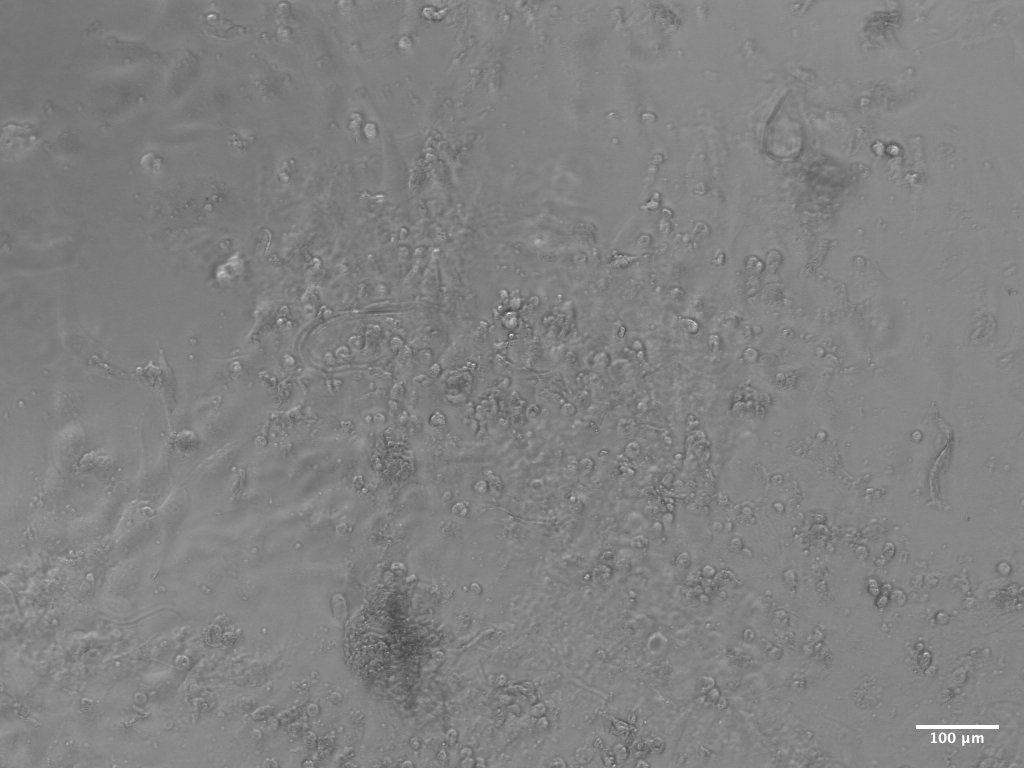
Figure S3.** Human umbilical vein endothelial cells (HUVECs) in 0.125% nanofibrillated cellulose hydrogel (NFCh) without conditioned media (CM) stimulation on day 4. Scale bar, 100 µm.

### 2.3 Stack images of the confocal microscopy

All the cells were imaged using confocal microscopy at ×10 magnification. Z-stack images were taken to ensure 3D cell organization was observed. In Figure S4 an example of the stack series in 0.8% 3D HUVEC monoculture is presented.


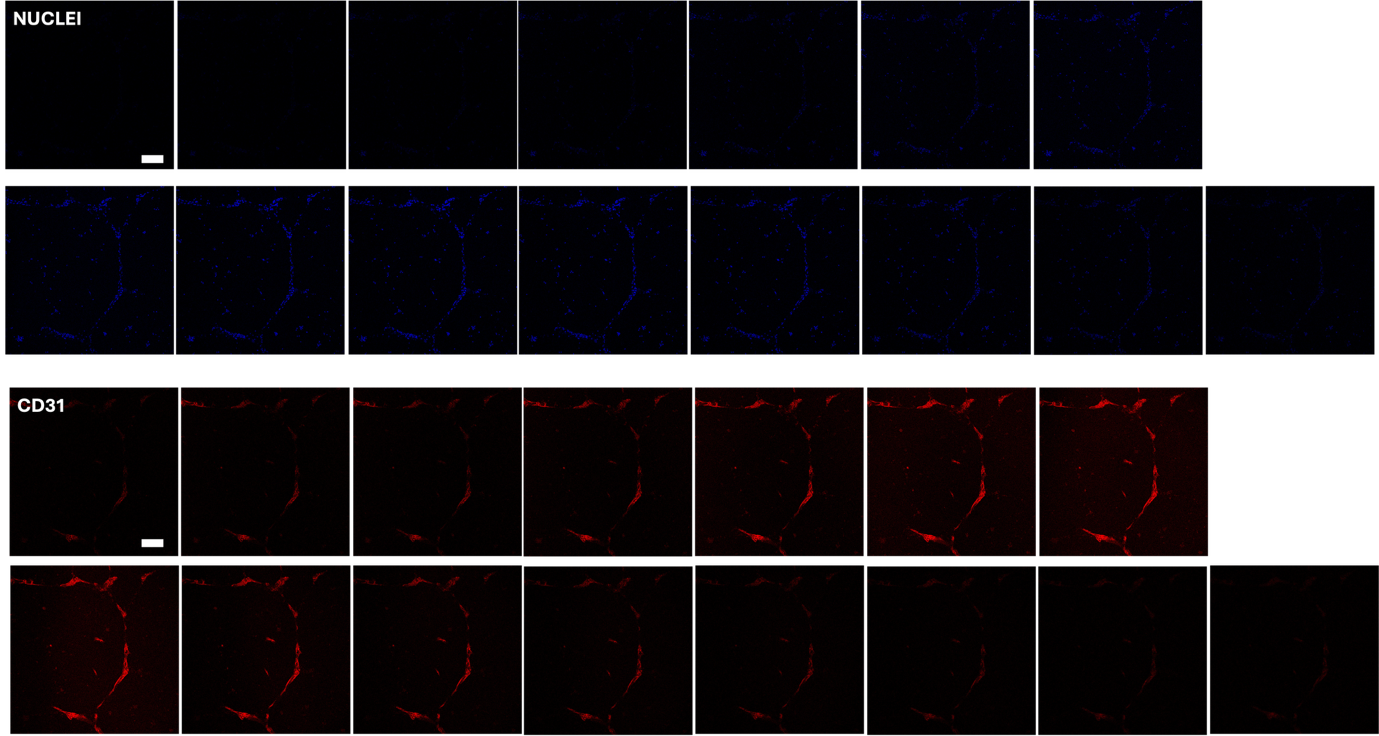
**Figure S4.** Stack images of the 0.8% three-dimensional (3D) human umbilical vein endothelial cell (HUVEC) monoculture. Scale bar 200 µm.

### 2.4 Fluorescent intensity of vWF and CD31 staining in co-culture systems

The fluorescent intensities of vWF and CD31 staining in the HUVEC–hASC co‑culture systems were evaluated (Figure S5). Regardless of the matrix used (0.125% NFCh, 1.5% NFCh, or Matrigel), vWF consistently exhibited higher fluorescence intensity than CD31, although with notably larger standard deviations.


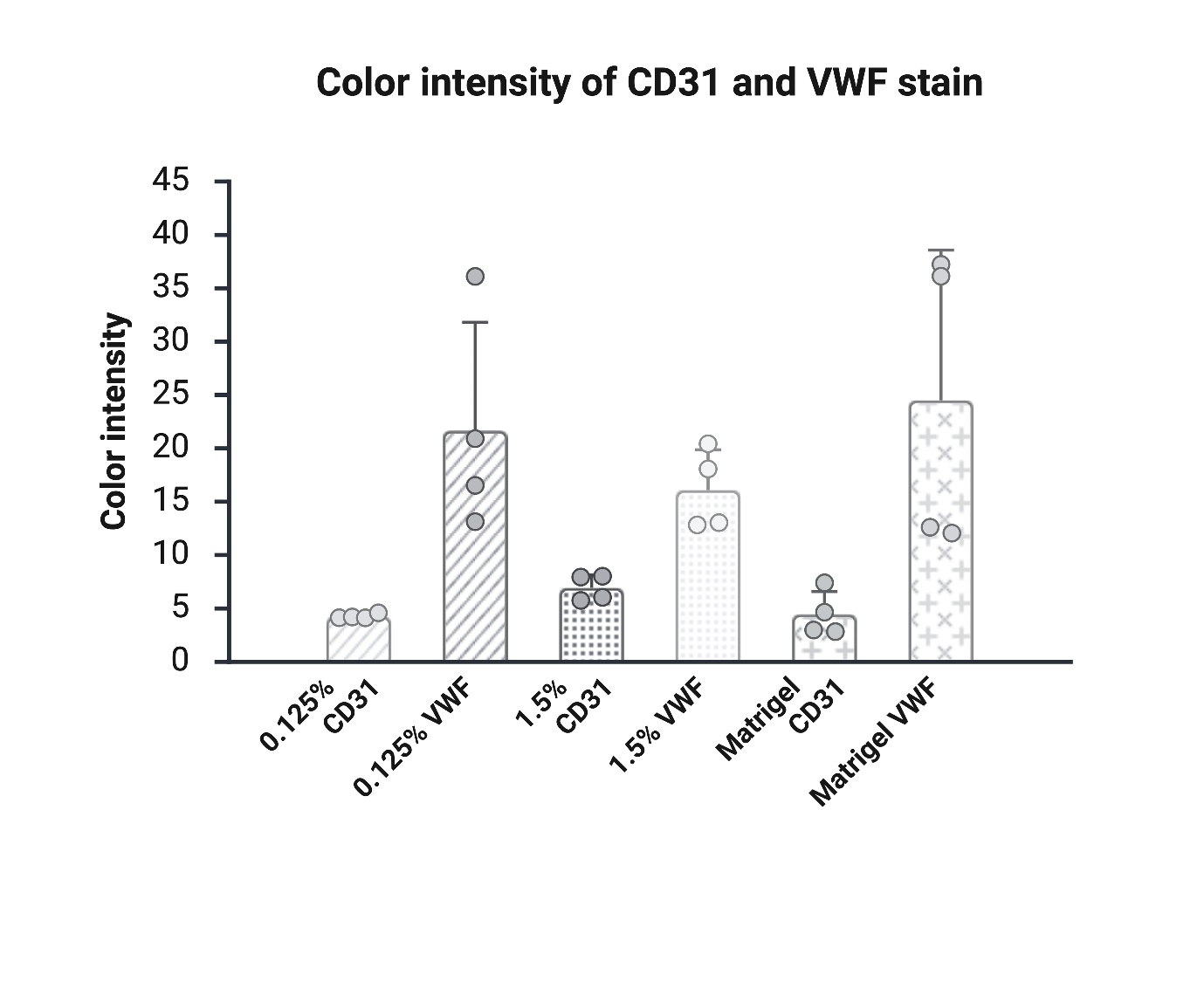


**Figure S5.** Fluorescent intensity of vWF and CD31 staining in human umbilical vein endothelial cell (HUVEC) and human adipose-derived stromal cell (hASC) co-cultures.

### 2.5 Treemap visualization of the total proteomics analyses

The mechanisms associated with the proteomic changes in HUVEC cell pellets between NFCh 1.5% and NFCh 0.125% cell cocultures with were visualized with a treemap (Figure S6). The treemap shows the Gene Ontology Biological Process (GOBP) terms associated with these changes (Figure S6). The GOBP terms were clustered by semantic similarity, which resulted in 9 major functional clusters.

*
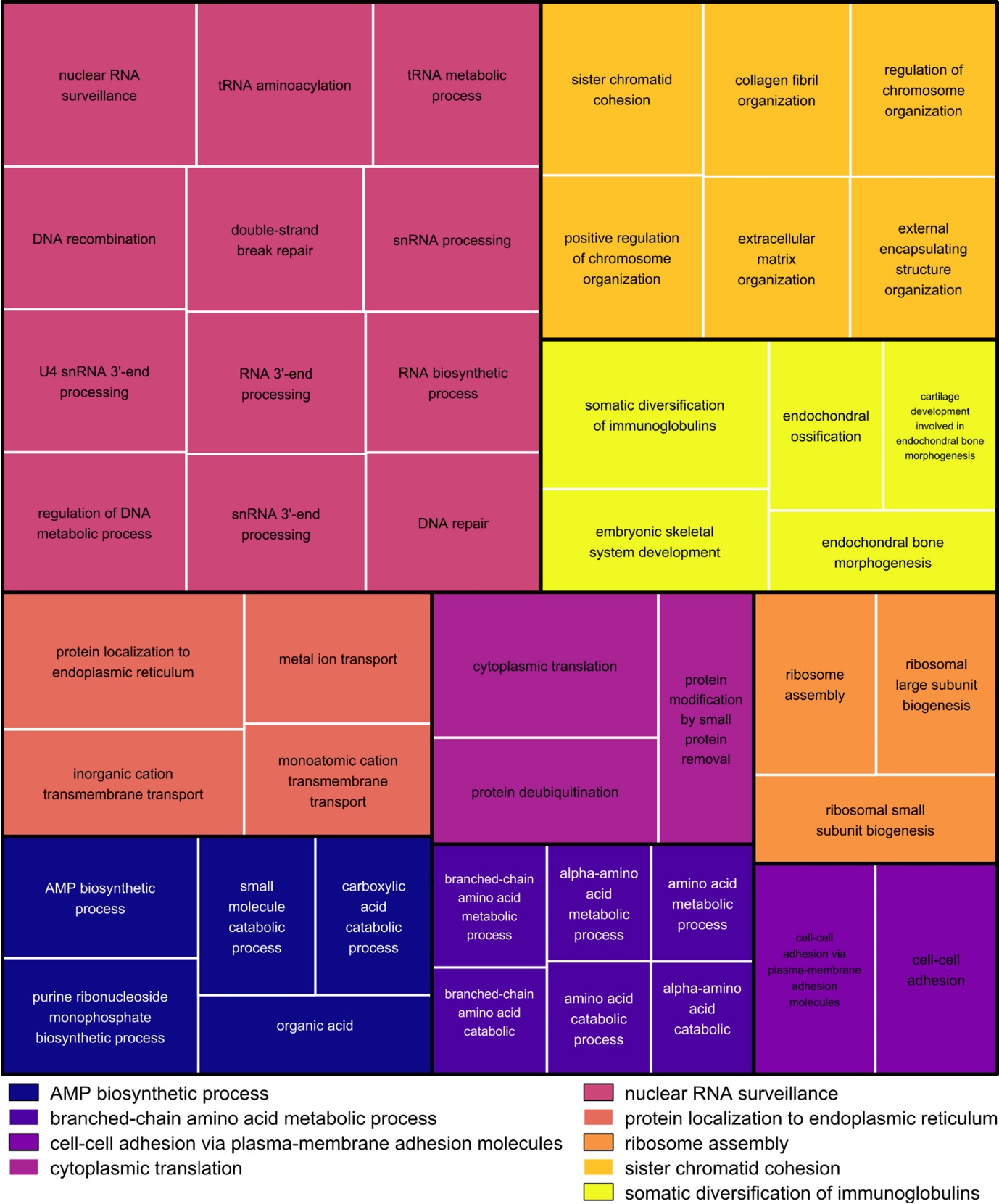
*

**Figure S6.** GOBP terms associated with changes in protein expression in cell pellets from nanofibrillated cellulose hydrogel (NFCh) 1.5% and NFCh 0.125% cell cocultures with human umbilical vein endothelial cells (HUVECs) and human adipose-derived stromal cells (hASCs). GOBP terms are clustered by semantic similarity into 9 groups and colour coded. Representative terms for each cluster are indicated in the legend.

**Table S1.** Used datasets in proteomics analyses and detected proteins in 0.125% NFCh and 1.5% NFCh samples. Protein analyses were carried out for both cell pellets and conditioned medium samples.

|  |  | **Cell pellet** | **Conditioned medium** |
| --- | --- | --- | --- |
|  | Members in total | **Detected in proteomic dataset** | **Detected in proteomic dataset** |
| **angiogenesis involved in wound healing** | **33** | **10** | **5** |
| **negative regulation of vasculature development** | **167** | **42** | **16** |
| **positive regulation of vasculature development** | **193** | **68** | **25** |
| **blood vessel morphogenesis** | **934** | **251** | **100** |
| **mesenchymal stem cell proliferation** | **12** | **4** | **2** |
| **stem cell proliferation** | **131** | **41** | **12** |
| **PanglaoDB pericyte** | **64** | **27** |  |

## References

1. Koivunotko E, Merivaara A, Niemelä A, Valkonen S, Manninen K, Mäkinen H, et al. Molecular Insights on Successful Reconstitution of Freeze-Dried Nanofibrillated Cellulose Hydrogel. ACS Appl Bio Mater. 2021;4(9).

2. Zudaire E, Gambardella L, Kurcz C, Vermeren S. A computational tool for quantitative analysis of vascular networks. PLoS One. 2011;6(11).

3. Koivunotko E, Snirvi J, Merivaara A, Harjumäki R, Rautiainen S, Kelloniemi M, et al. Angiogenic Potential of Human Adipose-Derived Mesenchymal Stromal Cells in Nanofibrillated Cellulose Hydrogel. Biomedicines. 2022;10(10).
